# Novel Cerium Phosphate–Citric Acid Nanocomposites for Biomedical Applications in Regenerative Medicine: In Vitro and In Vivo Study

**DOI:** 10.3390/ijms27167476

**Published:** 2026-08-21

**Authors:** Ekaterina V. Silina, Natalia E. Manturova, Elena L. Chuvilina, Akhmedali A. Gasanov, Olga I. Andreeva, Elena B. Artyushkova, Mikhail P. Gladchenko, Aleksandr V. Ivanov, Victor T. Dudka, Sergey Y. Mironov, Daria D. Tkachenko, Anatoly V. Skalny, Alexey A. Tinkov, Natalia Y. Tabachkova, Victor A. Stupin

**Affiliations:** 1 I.M. Sechenov First Moscow State Medical University (Sechenov University), Ministry of Health of the Russian Federation, Moscow 119991, Russia; a.olga@lanhit.ru (O.I.A.); daryatkch@mail.ru (D.D.T.); skalnyy_a_v@staff.sechenov.ru (A.V.S.); tinkov.a.a@gmail.com (A.A.T.); 2 Pirogov Russian National Research Medical University, Ministry of Health of the Russian Federation, Moscow 117997, Russiastvictor@bk.ru (V.A.S.); 3 LANHIT LLC, Moscow 105118, Russia; chuvilina.elena@lanhit.ru (E.L.C.); akhmedali@lanhit.ru (A.A.G.); 4 Kursk State Medical University, Ministry of Health of the Russian Federation, Kursk 305041, Russia; eartyushkova@mail.ru (E.B.A.); mgladchenko@yandex.ru (M.P.G.); ivanovav@kursksmu.net (A.V.I.); dudkavt@kursksmu.net (V.T.D.); mironovsyu@kursksmu.net (S.Y.M.); 5 Yaroslavl State University, Ministry of Science and Higher Education of the Russian Federation, Yaroslavl 150003, Russia; 6 National University of Science & Technology MISIS, Ministry of Science and Higher Education of the Russian Federation, Moscow 119049, Russia; ntabachkova@gmail.com

**Keywords:** nanoparticles, nanocomposite, cerium phosphate, cerium (III) orthophosphate, citric acid, citrate, morphology, TEM, X-ray diffraction, IR spectroscopy, antioxidants, redox activity, safety, wound healing, rats, mice, toxicology, toxicity, regenerative medicine

## Abstract

The aim of the study was to synthesize nanocomposites based on cerium orthophosphate stabilized with citrate at different ratios for biomedical applications, and to evaluate its safety in different animal species. Nanocomposites were synthesized using four different initial mass ratios of Ce(NO_3_)_3_ × 6H_2_O to citric acid: 1:5 (Ce:5CA), 1:3 (Ce:3CA), 1:1 (Ce:1CA), and 1:0.5 (Ce:0.5CA). The obtained products were characterized by a series of physicochemical methods including X-ray diffraction, transmission electron microscopy, and IR spectroscopy. Biomedical studies of the sols were performed to evaluate redox activity and their effects on the metabolism and proliferation of human fibroblasts. Safety studies were carried out in different animal species, including determination of the acute toxicity of the selected nanocomposite following application to intact skin and intragastric administration, as well as determination of subchronic toxicity using a model of acute experimental full-thickness skin wounds. According to the physicochemical studies, all synthesized nanocomposites contained nanocrystals with bimodal dimensions (width 3–10 nm, length 15–40 nm) and diffraction peaks characteristic of CePO_4_. IR spectroscopy demonstrated the formation of new chemical bonds. Antioxidant activity was observed for the Ce:3CA, Ce:1CA, and Ce:0.5CA samples, being most pronounced for Ce:1–0.5CA, whose antioxidant capacity exceeded that of ascorbic acid. In contrast, Ce:5CA exhibited pro-oxidant activity at all tested concentrations. Biocompatibility with human cells was demonstrated. Ce:0.5CA was identified as the most promising candidate for biomedical applications owing to its highest regenerative and antioxidant potential and its demonstrated safety in animals following topical and intragastric administration, as well as in wound models. Histomorphological examination of the internal organs supported the safety and potential of the developed nanocomposites for skin regeneration and wound healing.

## 1. Introduction

Rare earth metals have come to be regarded as key elements of the twenty-first century because of their potential for the development of novel materials, owing to their exceptional physical and chemical properties and their wide range of practical applications, including in biomedicine [1,2,3]. Cerium, the most abundant of all rare earth elements in the Earth’s crust, possesses a unique electronic configuration (4f^1^5d^1^6s^2^), the ability to reversibly transition between Ce^3+^ and Ce^4+^, and exhibits high biocompatibility and biological activity of its compounds, including those intended for regenerative medicine [1,2,3,4,5,6,7,8]. In recent years, rare earth orthophosphates have been extensively investigated because of the broad range of interesting properties they exhibit [9,10,11]. Cerium phosphate (CePO_4_) stands out among other orthophosphates because of its unique photophysical properties, including a high refractive index, photochemical stability, and low solubility [3,12,13,14,15].

CePO_4_ exists in two polymorphic forms: the low-temperature hydrated hexagonal rhabdophane phase and the high-temperature monoclinic monazite phase. The use of chemically stable monazite for biomedical applications has been mainly focused on the development of bioresorbable materials [3,16,17,18], whereas the more chemically active rhabdophane may be used to mimic multienzyme activity and for antibacterial and regenerative purposes [19,20,21,22].

Previous studies have shown that the morphology of the resulting CePO_4_ crystallites depends on the synthesis method [21,22,23,24,25,26]. The properties of the CePO_4_ nanoarchitecture strongly depend on its dimensionality, morphology, size uniformity, and crystallinity (the relationship between structure and morphology and the resulting properties), and these aspects are highly sensitive to the synthesis methodology employed [21,22,26,27,28]. Although nanosized rare earth orthophosphates such as CePO_4_ can be prepared by simple dissolution and coprecipitation methods, the controlled synthesis of these nanomaterials often requires the use of chelating or complexing additives, as well as strict control of the synthesis conditions, including the selection of precursors as sources of cerium and phosphate ions, their ratio, temperature, contact time, pH, precipitation conditions, and the order and rate of precursor addition.

It is well known that nanoparticles with high surface energy tend to agglomerate and form large aggregates. To prevent this, stabilizing agents are used to provide electrostatic or steric stabilization of nanoparticles by forming a protective layer that keeps the particles separated while preserving their nanoscale dispersion [29]. Stabilizing agents also regulate particle growth, making it possible to obtain particles of predetermined size and shape [29,30,31], and ensure the long-term stability of colloidal systems by preventing sedimentation and changes in the characteristics of nanomaterials over time.

Surfactants that provide steric and/or electrostatic stabilization [32], polymers that form shells around nanoparticles [33], ions and charged molecules (such as carboxyl or sulfate groups in organic compounds capable of creating an electrostatic barrier) [34], and ligand molecules that bind to the nanoparticle surface through donor–acceptor interactions, thereby stabilizing them [35], are commonly used as stabilizing agents in laboratory synthesis. The selection of biocompatible stabilizers for medical applications is often determined by the fact that some ligands act as metabolites in living organisms or exhibit their own biological activity. Citric acid, which participates in the tricarboxylic acid cycle and possesses significant antioxidant activity, is one of the most commonly used stabilizing excipients and is an example of such a ligand [36,37,38,39]. Citric acid, when added to the reaction medium as a stabilizing agent, is capable of influencing the morphology of the resulting cerium-containing compounds [29,40,41,42,43]. The use of citric acid as an additional component in the synthesis of nanosized cerium-containing materials (primarily CeO_2_) makes it possible to carry out synthesis at low temperatures and ensures homogeneity in the distribution of nanoparticles possessing regenerative, antioxidant, and antimicrobial properties [38,44]. At the same time, in systems containing citric acid and salt solutions, dissociation and hydrolysis processes lead to complex equilibria that directly depend on the concentrations of the starting substances and the pH of the solution [45].

The development of biomedical products based on cerium phosphate nanoparticles is a novel and promising field. The main beneficial biomedical properties of these nanomaterials are biocompatibility, regenerative potential, and, according to current concepts of cell life and death, antioxidant (redox) activity. The redox activity of cerium compounds is associated with the Ce^3+^ ↔ Ce^4+^ electronic transition, which, in turn, contributes to antimicrobial activity and makes a substantial contribution to the high regenerative potential of biomedical products [46,47,48]. Nanosized cerium phosphates have demonstrated biocompatibility and novel beneficial regenerative properties, as confirmed in experiments using both cell cultures and in vivo models. Recent studies have shown that nanosized cerium phosphate samples stimulate the proliferation of human mesenchymal stem cells, fibroblasts, and keratinocytes while simultaneously exhibiting antioxidant properties [22,49,50]. A terbium-doped cerium phosphate-based hydrogel contributed to the reduction in oxidative stress, enhanced fibroblast proliferation and differentiation in vitro, and enhanced wound regeneration in vivo [51]. At the same time, the redox activity of CePO_4_ depends on the phase and the environment, that is, cerium phosphates are capable of exhibiting both antioxidant and prooxidant properties [48,49,50,52,53].

Thus, the development of novel drugs based on cerium phosphate nanocomposites represents a new and promising field. The choice of a synthesis method for cerium phosphate-based nanocomposites with desired beneficial biomedical properties depends on the required characteristics of the final product, including size, morphology, crystallinity, as well as production conditions and process scalability, which necessitates fundamental interdisciplinary research.

The aim of the study was to develop cerium phosphate–citrate nanocomposites for biomedical applications.

To achieve this aim, the following objectives were established:

1. To synthesize nanosized cerium (III) orthophosphate + citric acid composites with different initial ratios (from 1:5 to 1:0.5).
2. To perform physicochemical characterization of the synthesized nanocomposites using transmission electron microscopy (TEM), X-ray diffraction (XRD), and Fourier-transform infrared (FTIR) spectroscopy.
3. To investigate the redox activity of cerium orthophosphate–citrate nanocomposites over a wide concentration range using chemiluminescence.
4. To perform in vitro biomedical studies of CePO_4_–CA nanocomposite sols using different cell lines to determine their biocompatibility and their ability to stimulate the metabolism and proliferation of human fibroblasts.
5. To select the best nanocomposite and evaluate its safety in vivo by determining acute toxicity following application to intact skin and intragastric administration, as well as subchronic toxicity using a model of experimental acute full-thickness skin wounds.

## 2. Results

### 2.1. Results of the Evaluation of the Physicochemical Properties of the Synthesized Cerium (III) Orthophosphate–Citric Acid Nanocomposites

#### 2.1.1. Transmission Electron Microscopy

**Figure 1:**
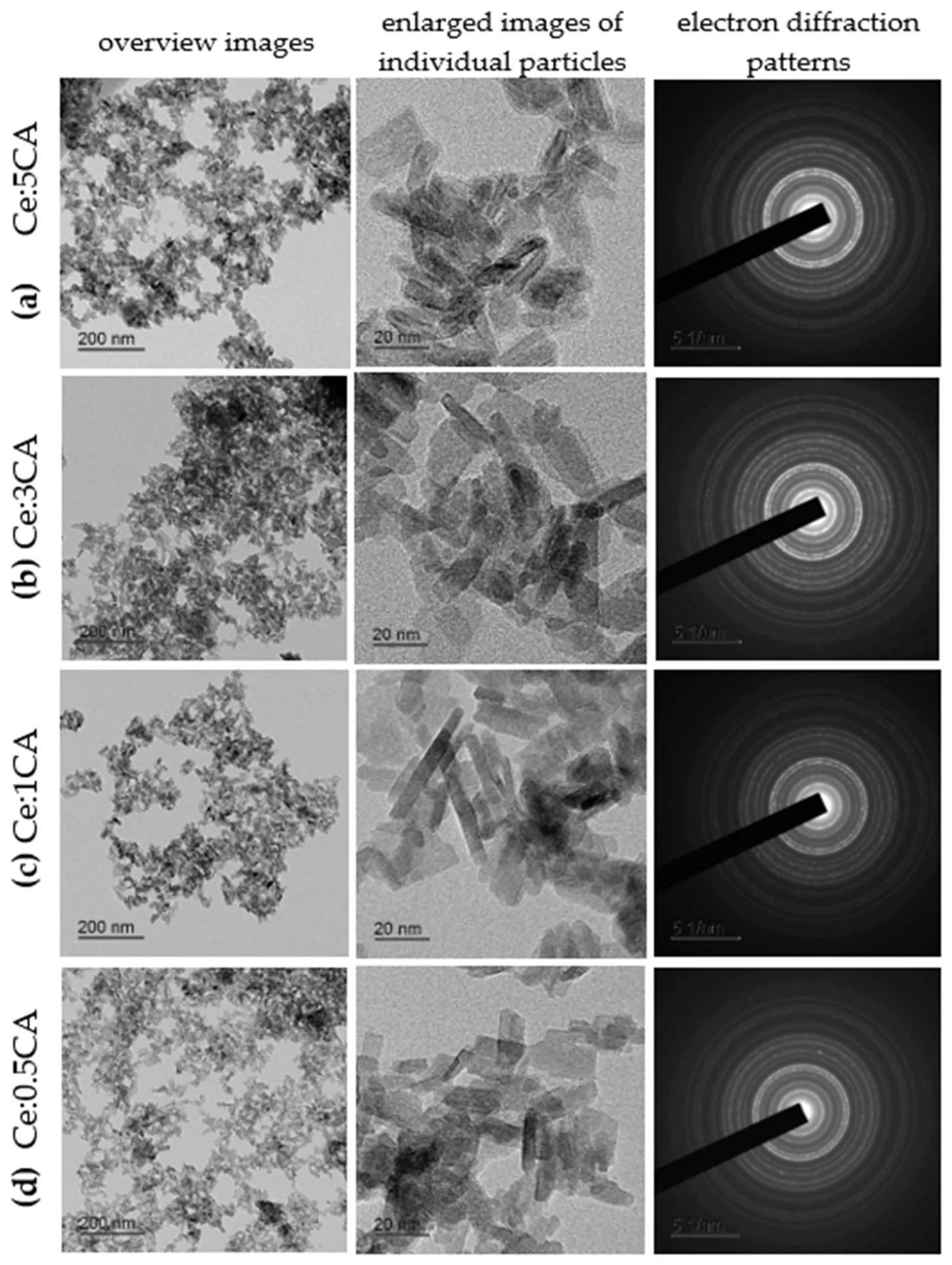
Images of the particles in the CePO_4_–citric acid nanocomposites, overview images (**left**), enlarged images of individual particles (**middle**), and electron diffraction patterns (**right**). (**a**) Ce:5CA; (**b**) Ce:3CA; (**c**) Ce:1CA; (**d**) Ce:0.5CA.

**Figure 2:**
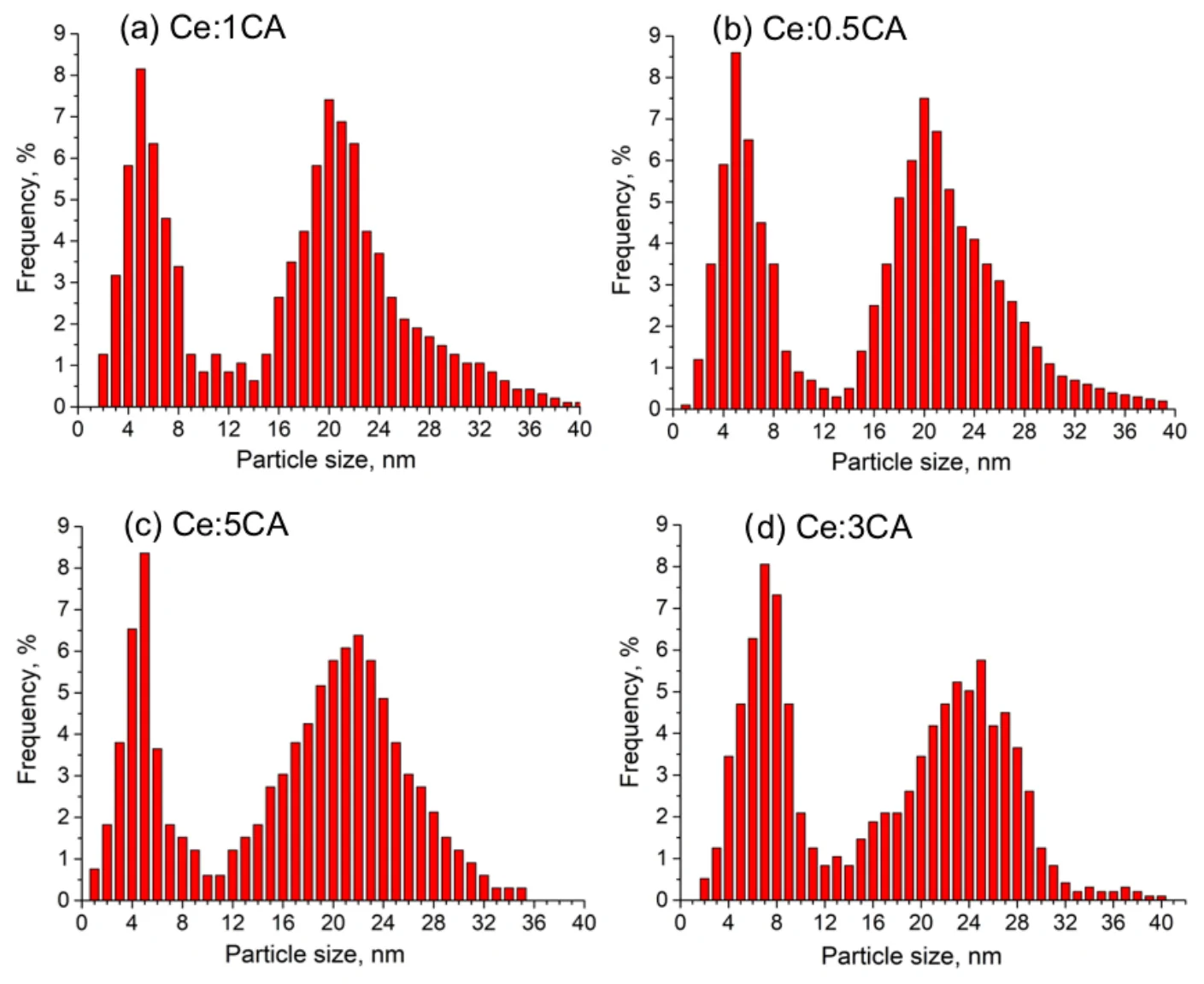
Particle size distribution of the CePO_4_–citric acid nanocomposites (nm). (**a**) Ce:1CA; (**b**) Ce:0.5CA; (**c**) Ce:5CA; (**d**) Ce:3CA.

According to the TEM data, in all studied samples, the average size of individual cerium phosphate particles exhibited a bimodal distribution with well-defined maxima. The degree of agglomeration of the investigated powders differed and correlated with the initial Ce ratios. Figure 1 shows TEM images of the particles, whereas Figure 2 presents the corresponding particle size distributions.

In the Ce:5CA sample (Figure 1a), the powder particles were weakly agglomerated, and individual nanoparticles randomly arranged within the agglomerates were clearly distinguishable. The positions of the reflections in the selected-area electron diffraction patterns corresponded to the CePO_4_ phase. The particles were anisotropic. The particle size distribution (Figure 2a) showed that the particle width varied only slightly, mainly from 2 to 9 nm, with the distribution maximum at approximately 4–5 nm. The particle length varied from 5 to 35 nm, with the distribution maximum at 19–24 nm. The median size of the Ce:5CA cerium phosphate nanoparticles was 5 × 22 nm.

Figure 1b shows TEM images of the particles in the Ce:3CA sample. The particles were agglomerated (agglomerates ranging from 30 to 200 nm were observed, consisting of randomly packed fine particles). The reflections in the electron diffraction pattern corresponded to the CePO_4_ phase. The particles had an anisotropic shape. High-resolution images clearly revealed atomic lattice fringes in nearly all particles. Figure 2b presents the particle size distribution of the Ce:3CA sample. The particle length ranged mainly from 10 to 40 nm, with a maximum at 23–25 nm, whereas the particle width ranged from 3 to 12 nm, with a distribution maximum at 6–8 nm. The median particle size of the Ce:3CA nanoparticles was 7 × 25 nm.

Figure 1c,d presents TEM images of the particles in the Ce:1CA and Ce:0.5CA samples. As can be seen, the Ce:1CA sample was less agglomerated. The positions of the reflections in the electron diffraction pattern corresponded to the CePO_4_ phase. The particles had an elongated shape, with their length exceeding their width by a factor of 2–3. High-resolution images clearly revealed atomic lattice fringes. A similar pattern was observed for the Ce:0.5CA sample, in which the particles also exhibited an anisotropic shape.

Figure 2c shows the particle size distribution of the Ce:1CA sample. The particle width ranged from 2 to 10 nm, with a pronounced maximum at 5 nm. The particle length ranged from 10 to 40 nm, with the distribution maximum at approximately 20–22 nm. Similar characteristics were observed for the Ce:0.5CA sample (Figure 2d): the particle width ranged from 2 to 12 nm, the first distribution maximum was at 5 nm, and the second distribution maximum was at 20 nm.

Thus, all synthesized cerium phosphate nanoparticles stabilized with citric acid were characterized by an elongated particle shape, particle anisotropy, and a bimodal crystallite size distribution. The particle sizes varied from 2 to 40 nm, with maxima at 4–6 nm (width) and 18–26 nm (length). No clear dependence of particle size on the concentration of citric acid in the system was established. However, it is fundamentally important that the addition of citric acid at the studied concentrations ensured the formation of consistently small cerium phosphate particles.

According to the TEM data, lower nanoparticle agglomeration and higher crystallinity (the presence of atomic lattice fringes) were observed for the Ce:1CA and Ce:0.5CA samples. Thus, it can be assumed that an initial cerium-to-citrate ratio of 1:0.5–1 is optimal.

#### 2.1.2. X-Ray Diffraction Analysis

**Figure 3:**
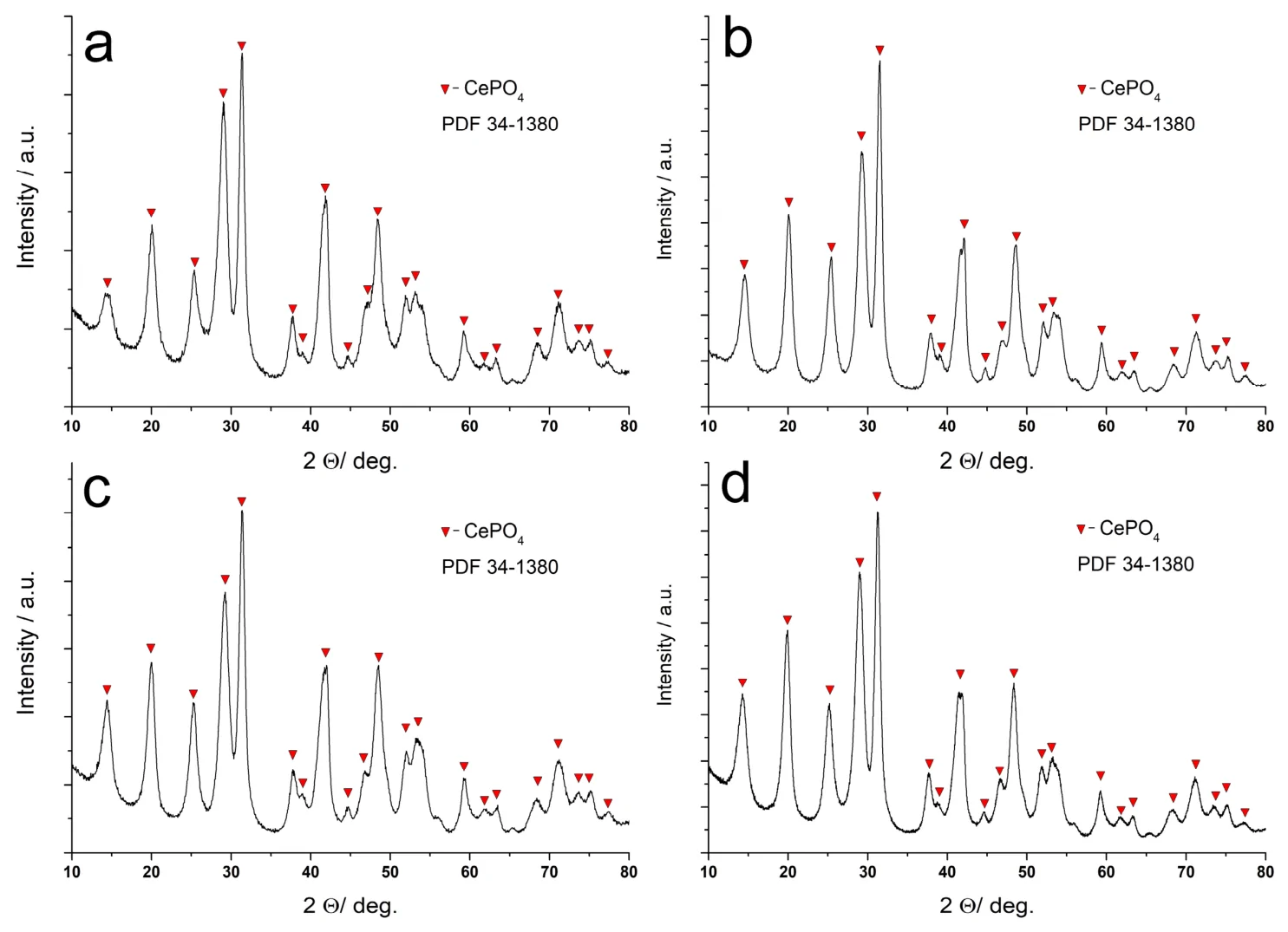
X-ray diffraction patterns of nanocrystalline cerium (III) phosphate samples synthesized by the precipitation method at different initial cerium nitrate:citric acid ratios. (**a**) Ce:5CA; (**b**) Ce:3CA; (**c**) Ce:1CA; (**d**) Ce:0.5CA.

To determine the phase composition of the synthesized nanomaterials, X-ray diffraction (XRD) analysis was performed for all synthesized powders. The XRD patterns of the samples are shown in Figure 3.

All diffraction patterns demonstrated that the powders consisted of single-phase cerium (III) phosphate with the rhabdophane structure, which is consistent with the TEM data. The sizes of the coherent scattering domains of the CePO_4_ powders, determined from the widths of the diffraction peaks, were 15–20 nm.

It was established that varying the reagent ratio affected the intensity and width of the diffraction peaks. Broader peaks indicate smaller crystallite sizes (nanoscale dimensions). The diffraction peaks of the samples are referred to the reference file PDF 34-1380 CePO_4_, which corresponds to the crystalline phase of hexagonal cerium phosphate.

In all diffraction patterns, the major peaks coincided with the reference peaks, confirming the formation of cerium phosphate at all investigated reagent ratios.

#### 2.1.3. FTIR Spectroscopy Results

To confirm the formation of cerium phosphate nanocomposites in the Ce–CA systems (1:5–1:0.5), the samples were analyzed by FTIR spectroscopy, which makes it possible to evaluate the formation of new bonds based on shifts in the absorption bands of characteristic functional groups compared with the starting materials.

Starting materials:

• The FTIR spectrum of pure citric acid contains absorption bands associated with the stretching and bending vibrations of O–H, C–H, and C=O bonds. In the region of 3600–3200 cm^−1^, a broad and intense band is observed, corresponding to the stretching vibrations of hydroxyl (–OH) groups involved in hydrogen bonding. The bending vibrations of hydroxyl (–OH) groups appear in the regions of 1450–1400 cm^−1^ and 1300–1200 cm^−1^. The carboxyl (COOH) groups produce a strong absorption band in the region of 1760–1700 cm^−1^, corresponding to the stretching vibration of the C=O bond. In the region of 1300–1000 cm^−1^, absorption bands associated with the stretching vibrations of C–O bonds in carboxyl and hydroxyl groups are observed.
• The FTIR spectrum of CePO_4_ is characterized by the presence of absorption bands corresponding to the vibrations of P–O bonds in the phosphate ion. The principal bands are typically observed in the regions of 900–1200 cm^−1^ and 400–600 cm^−1^.

**Figure 4:**
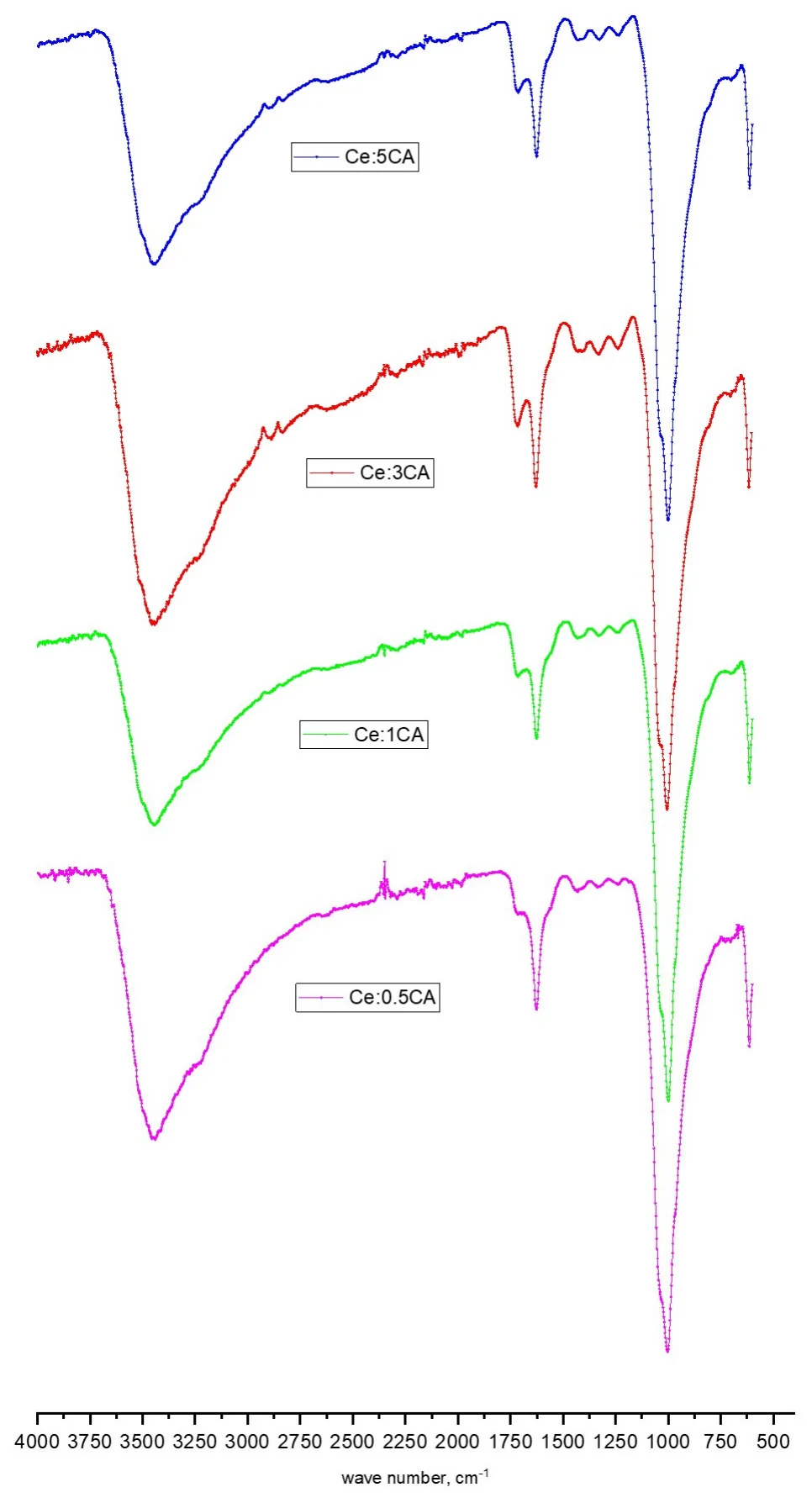
FTIR spectra of samples prepared with different reactant ratios (from **top** to **bottom**): Ce:CA = 1:5, 1:3, 1:1, and 1:0.5.

The FTIR spectra of the non-calcined samples (Figure 4) are characterized by the following principal absorption bands:

• The most intense peak is located in the region of approximately 1000–1100 cm^−1^, corresponding to the asymmetric stretching vibrations of the P–O bond;
• Weaker peaks or shoulders near 600 cm^−1^ and 530 cm^−1^ are most likely associated with O–P–O bond vibrations;
• The presence of a citrate coating is indicated by peaks corresponding to the carbonyl group (distinct peaks in the region of 1600–1750 cm^−1^ assigned to the C=O bond) and by the peak near 1605 cm^−1^, assigned to vibrations of the coordinated COO^−^ group;
• Weak peaks at approximately 2920 and 2968 cm^−1^ correspond to the stretching vibrations of the aliphatic CH_3_ group of citric acid;
• Hydroxyl groups and water are represented by a broad band in the region of 3400–3500 cm^−1^, corresponding both to the stretching vibrations of the OH groups of adsorbed water and to surface hydroxyl groups.

As the component ratio changes (from 1:5 to 1:0.5), the relative intensity of the peaks associated with citric acid (particularly the COO^−^ bands near 1600 cm^−1^) decreases and may shift, reflecting the amount of organic coating on the phosphate surface. The unchanged peaks near approximately 1000 and 600 cm^−1^, corresponding to the cerium phosphate structure, indicate the stability of the main inorganic phase.

Comparison of the FTIR spectra of cerium phosphate samples coated with citric acid at ratios of 1:5 (blue spectrum) and 1:0.5 (purple spectrum) reveals structural changes associated with the concentration of the organic layer.

(a) The most significant changes are observed in the spectral region corresponding to citric acid:
  • Carbonyl region (~1600–1750 cm^−1^): The peaks corresponding to the asymmetric stretching vibrations of the COO^−^ groups (~1630 cm^−1^) are most intense in the Ce:5CA sample. As the ratio changes to Ce:0.5CA, the intensity of these peaks decreases markedly, indicating a reduction in the amount of adsorbed citric acid on the nanoparticle surface.
  • Aliphatic C–H groups (~2850–2950 cm^−1^): The weak peaks in this region (symmetric and asymmetric stretching vibrations) become almost imperceptible when the citric acid content is reduced to the 1:0.5 ratio, confirming the formation of a thinner organic layer.
(b) Phosphate anion:
  • Asymmetric P–O stretching vibrations: The peak in the region of 1000–1100 cm^−1^ is the most intense in all samples. However, in the Ce:0.5CA sample this peak appears sharper and more pronounced relative to the citric acid absorption bands, since the cerium phosphate signal is practically not masked by the organic layer.
  • Bending vibrations (~530–600 cm^−1^)*:* These bands remain unchanged, confirming the preservation of the crystalline structure of cerium phosphate.
(c) Hydroxyl groups and water:
  • Region (~3400–3500 cm^−1^)*:* A broad band corresponding to the stretching vibrations of water and hydroxyl groups is present in all samples.

To understand the effect of heat treatment on the physicochemical properties of the nanocomposites, FTIR spectra were obtained for calcined samples with the limiting cerium-to-citric acid ratios of 1:5 and 1:0.5 (identical heat-treatment conditions: 200 °C for 2 h).

**Figure 5:**
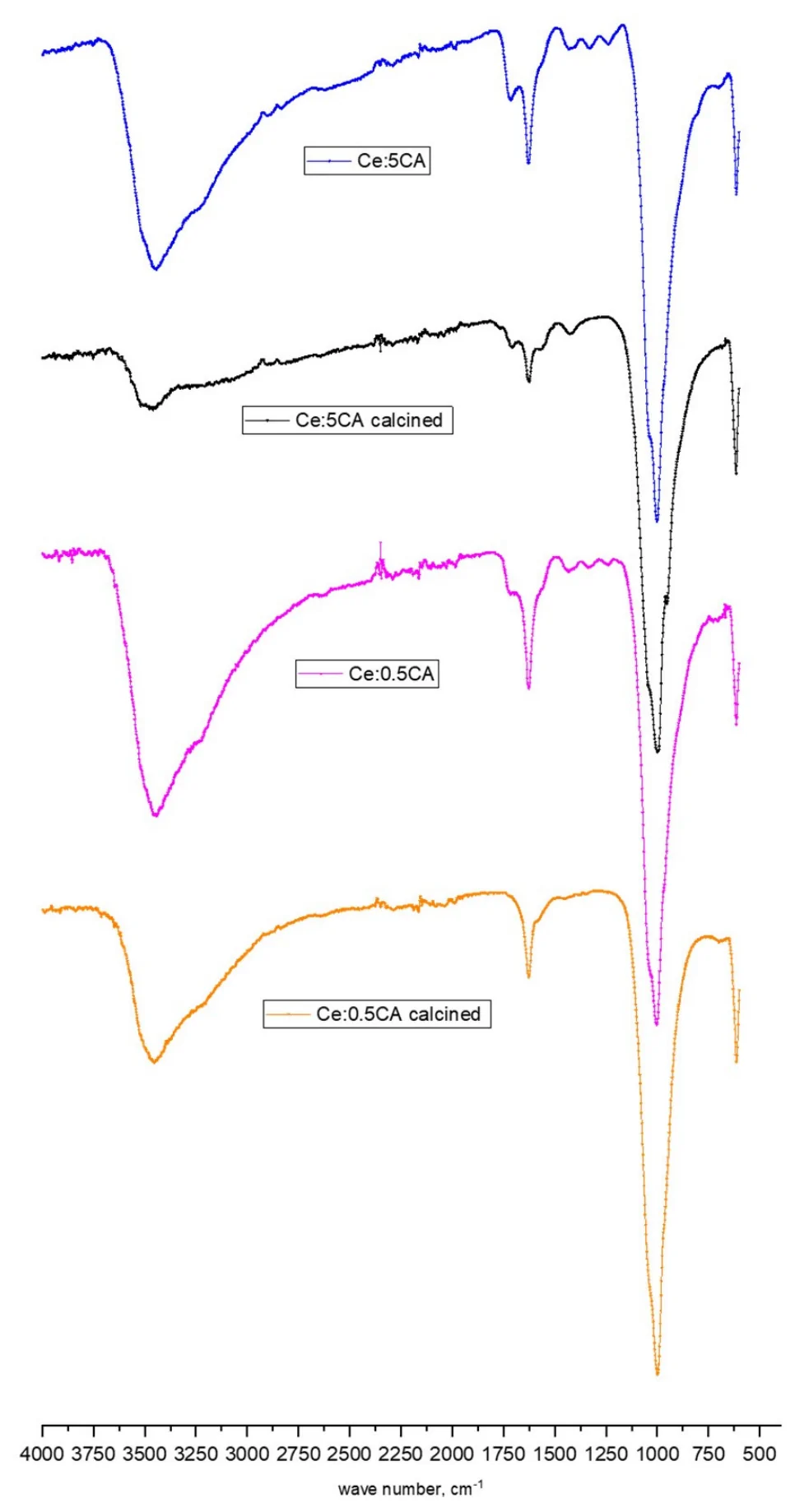
Comparison of the FTIR spectra of calcined and non-calcined samples. From top to bottom: 1:5, calcined 1:5, 1:0.5, and calcined 1:0.5.

The FTIR spectra of the calcined samples with the limiting reagent ratios (Figure 5) showed the following:

Citric acid carboxyl group absorption region (1700–1600 cm^−1^): In both calcined samples, the intensity of the peak near 1620 cm^−1^ (C=O bond) was significantly reduced compared with the corresponding non-calcined samples, confirming the decomposition of citric acid during calcination. However, this peak remained slightly more pronounced in the Ce:5CA sample (black curve), which may indicate the presence of a small amount of residual citric acid due to its initially high concentration. Thus, the sample with this component ratio did not decompose completely under the selected heat-treatment conditions.

Phosphate anion absorption region (1100–950 cm^−1^): In the calcined Ce:5CA sample (black curve), the phosphate stretching vibration peak appears sharper and narrower. This may indicate a higher degree of crystallinity and lattice ordering when a larger excess of citrate is used as a nanoparticle growth stabilizer. In the Ce:0.5CA sample (orange curve), the peak is broader, which is often characteristic of less homogeneous or smaller crystals.

Comparison of the spectra of the calcined and non-calcined samples with a 1:0.5 ratio shows that the absorption bands characteristic of citric acid are absent in the calcined sample. The spectrum of the calcined sample contains only the absorption bands of cerium phosphate. Thus, at a low citric acid content, the selected heat-treatment conditions were sufficient for complete decomposition of the nanocomposite.

The absorption bands for pure citric acid, nanosized cerium phosphate, and selected samples, together with their assignments, are presented in Table 1.

#### 2.1.4. Graphical Representation of the Synthesized CePO_4_–Citrate Nanocomposites

**Figure 6:**
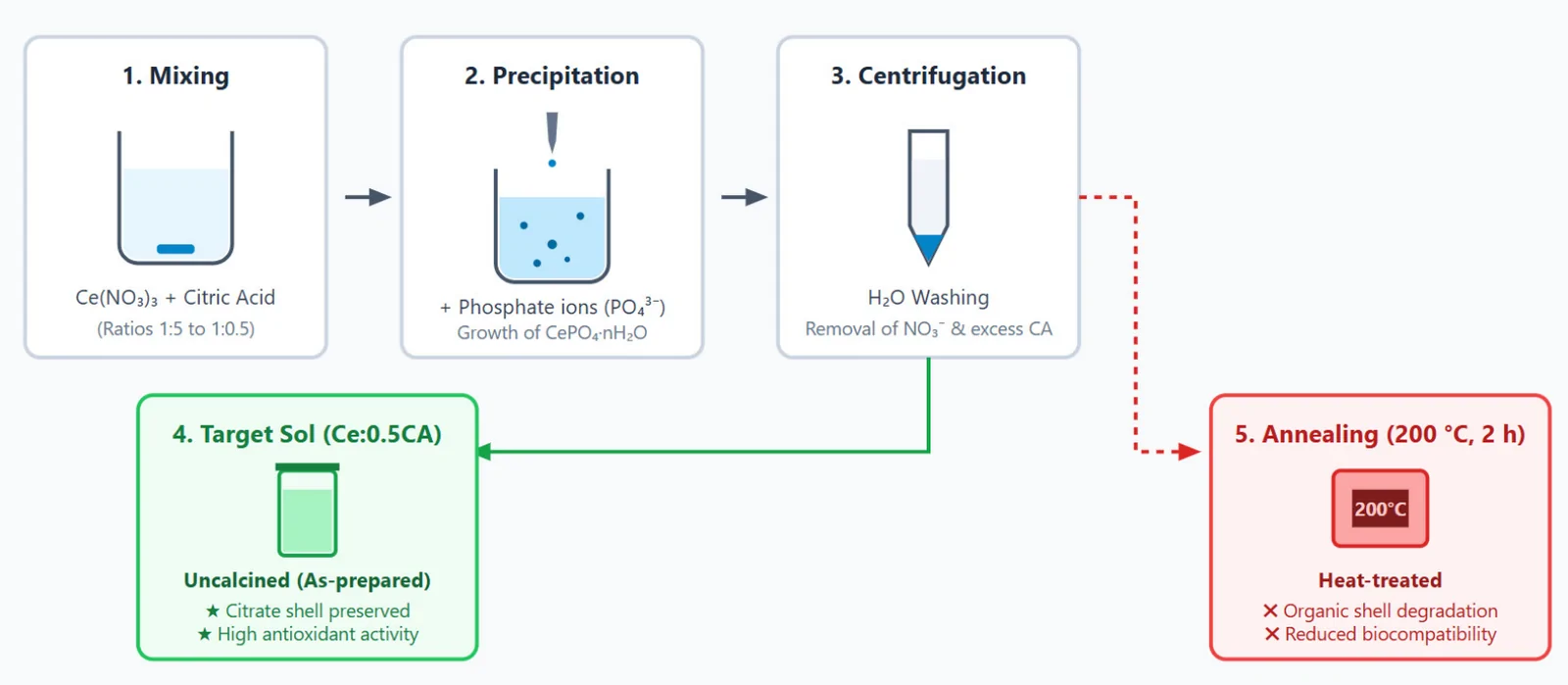
Synthesis and processing scheme of the CePO_4_–CA nanocomposites.

Based on the obtained data, we can propose a graphical representation of the resulting CePO_4_–Citrate nanocomposite. Figure 6 shows a scheme and result of the nanocomposites synthesis process.

### 2.2. Results of the Redox Activity Study

The study demonstrated that the Ce:0.5CA, Ce:1CA, and Ce:3CA samples exhibited dose-dependent antioxidant activity, whereas the Ce:5CA sample, in contrast, exhibited statistically significant dose-dependent prooxidant activity (Table 2).

The most pronounced redox activity was observed for the samples at a concentration of 10^−3^ M. The integral chemiluminescence intensity (S) in the presence of ascorbic acid (reference antioxidant) at a concentration of 10^−3^ M was, on average, 1.21-fold lower than that of the control (*p* < 0.05), while the maximum chemiluminescence intensity (Max) was 1.80-fold lower (*p* < 0.05).

**Figure 7:**
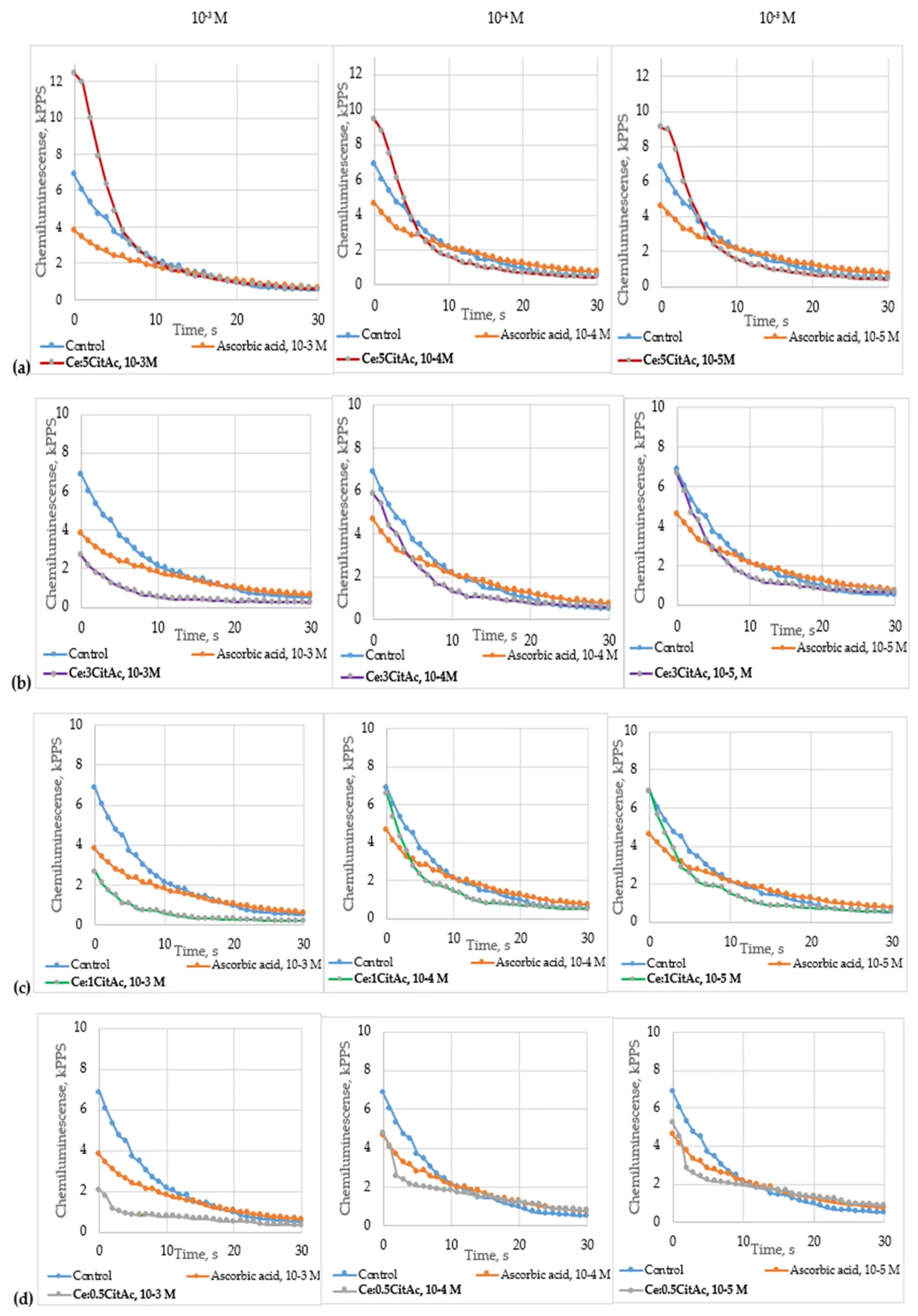
Kinetic profile of chemiluminescence in the reference experiment (control, blue line), in experiments with equal concentrations of antioxidant ascorbic acid (orange line), and with CePO_4_–CA nanocomposites at concentrations of 10^−3^ M (**left**), 10^−4^ M (**middle**), 10^−5^ M (**right**): (**a**) Ce:5CA; (**b**) Ce:3CA; (**c**) Ce:1CA; (**d**) Ce:0.5CA. The figures show the first 30 s of the reaction as the most demonstrative.

The Ce:5CA sample (Figure 7a) at a concentration of 10^−3^ M exhibited the most pronounced prooxidant activity. The S value increased significantly, reaching a level 1.33-fold higher than that of the control (*p* < 0.05) and 1.61-fold higher than that observed in the presence of 10^−3^ M ascorbic acid (*p* < 0.05). At the same time, the maximum chemiluminescence intensity was 1.81-fold higher than that of the control (*p* < 0.05) and 3.26-fold higher than that observed with 10^−3^ M ascorbic acid (*p* < 0.05).

The Ce:3CA (Figure 7b), Ce:1CA (Figure 7c), and Ce:0.5CA (Figure 7d) samples at a concentration of 10^−3^ M exhibited pronounced antioxidant activity. The integral chemiluminescence intensity was on average 2.50–2.71-fold lower than that of the control (*p* < 0.05) and 2.13–2.20-fold lower than that observed with ascorbic acid at the same concentration (*p* < 0.05). In addition, the maximum CL intensity in the control was, on average, 2.58-fold, 2.58-fold, and 3.33-fold higher than those observed for the Ce:3CA (*p* < 0.05), Ce:1CA (*p <* 0.05), and Ce:0.5CA (*p* < 0.05) samples, respectively; and 1.47-fold, 1.43-fold, and 1.85-fold higher than that observed with ascorbic acid, respectively. These findings indicate that the non-calcined CePO_4_:0.5–3CA nanocomposites added to the reaction system at a concentration of 10^−3^ M possess a greater antioxidant capacity. Although no statistically significant differences in antioxidant activity were observed among these three samples at a concentration of 10^−3^ M, the lowest chemiluminescence level was recorded for the Ce:0.5CA sample, which may be associated with the most efficient scavenging of reactive oxygen species.

Nanocomposites added to the experimental system at lower concentrations (10^−4^–10^−5^ M) exhibited less pronounced redox activity than those at 10^−3^ M; however, the direction of their pro- and antioxidant effects remained unchanged. Ce:5CA retained its prooxidant activity, whereas the Ce:3CA, Ce:1CA, and Ce:0.5CA samples exhibited antioxidant activity at a concentration of 10^−4^ M (Figure 7b–d).

The Ce:5CA sample was the only sample that demonstrated prooxidant activity not only at 10^−3^ M but also at concentrations of 10^−4^ and 10^−5^ M (Figure 7a). The integral chemiluminescence intensity was higher than that of the control and higher than that observed with ascorbic acid at the corresponding concentrations. At concentrations of 10^−4^ and 10^−5^ M, the maximum CL intensity was, on average, 1.38-fold and 1.32-fold higher than that of the control (*p* < 0.05), respectively, and 2.03-fold and 1.98-fold higher than that observed with ascorbic acid at the same concentrations.

Nanoparticles of Ce:3CA at a concentration of 10^−4^ M also demonstrated significant antioxidant activity compared with both the control (*p* < 0.05) and ascorbic acid. However, at 10^−5^ M, the ChL parameters were comparable to the control, whereas the maximum ChL intensity was significantly higher than that of the ascorbic acid group (*p* < 0.05), indicating the absence of antioxidant activity at high dilution.

The Ce:1CA nanocomposite sol at concentrations of 10^−4^–10^−5^ M also lost its advantage over ascorbic acid, and at 10^−5^ M the ChL parameters were comparable to those of the control.

For the Ce:0.5CA sample, antioxidant activity was maintained over a broad concentration range down to 10^−5^ M, although it was less pronounced than at 10^−3^ M. Even at 10^−4^–10^−5^ M, the maximum ChL intensity values of Ce:0.5CA were comparable to those of ascorbic acid at the same concentrations, while the S value at 10^−4^ M was on average 1.1-fold lower than that of ascorbic acid, indicating greater antioxidant activity.

Thus, the Ce:0.5CA, Ce:1CA, and Ce:3CA samples exhibited dose-dependent antioxidant activity, which was most pronounced at a concentration of 10^−3^ M and exceeded the antioxidant activity of ascorbic acid at the same concentration by 1.5–2.2-fold. In contrast, the Ce:5CA nanocomposite exhibited significant dose-dependent prooxidant activity, with the greatest effect observed at a concentration of 10^−3^ M.

### 2.3. Results of the Biological Activity Evaluation of Cerium Phosphate Nanocomposites in a Fibroblast Cell Line

**Figure 8:**
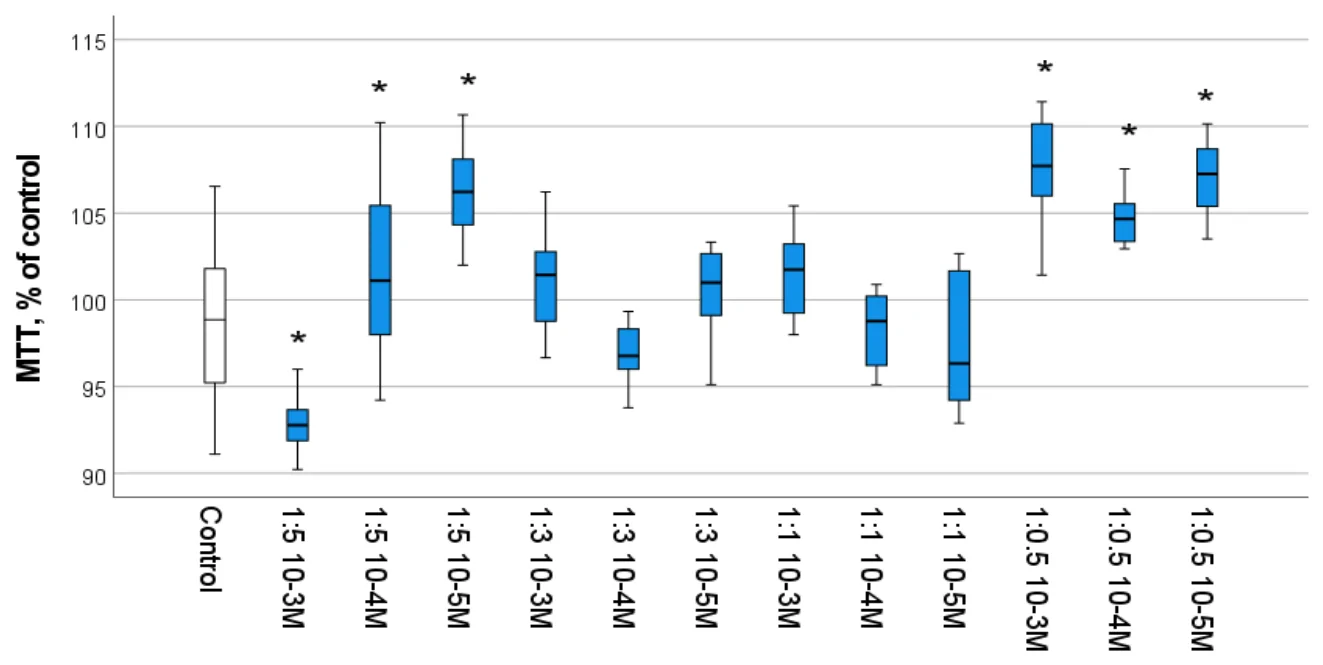
Effect of CePO_4_+CA nanocomposites with different reagent ratios on the metabolic activity of human BJ TERT fibroblasts in the MTT assay (*—*p*-value < 0.005, significant difference compared with the control).

According to the results of the MTT assay, it was established that stimulation of fibroblast metabolism occurs predominantly with the smallest nanoparticle shell at all investigated concentrations; in the Ce:0.5CA sample, the average OD value was 105–108% relative to the control (*p* < 0.05), as well as at the maximum CA content (1:5) at concentrations of 10^−4^–10^−5^ M (Figure 8).

**Figure 9:**
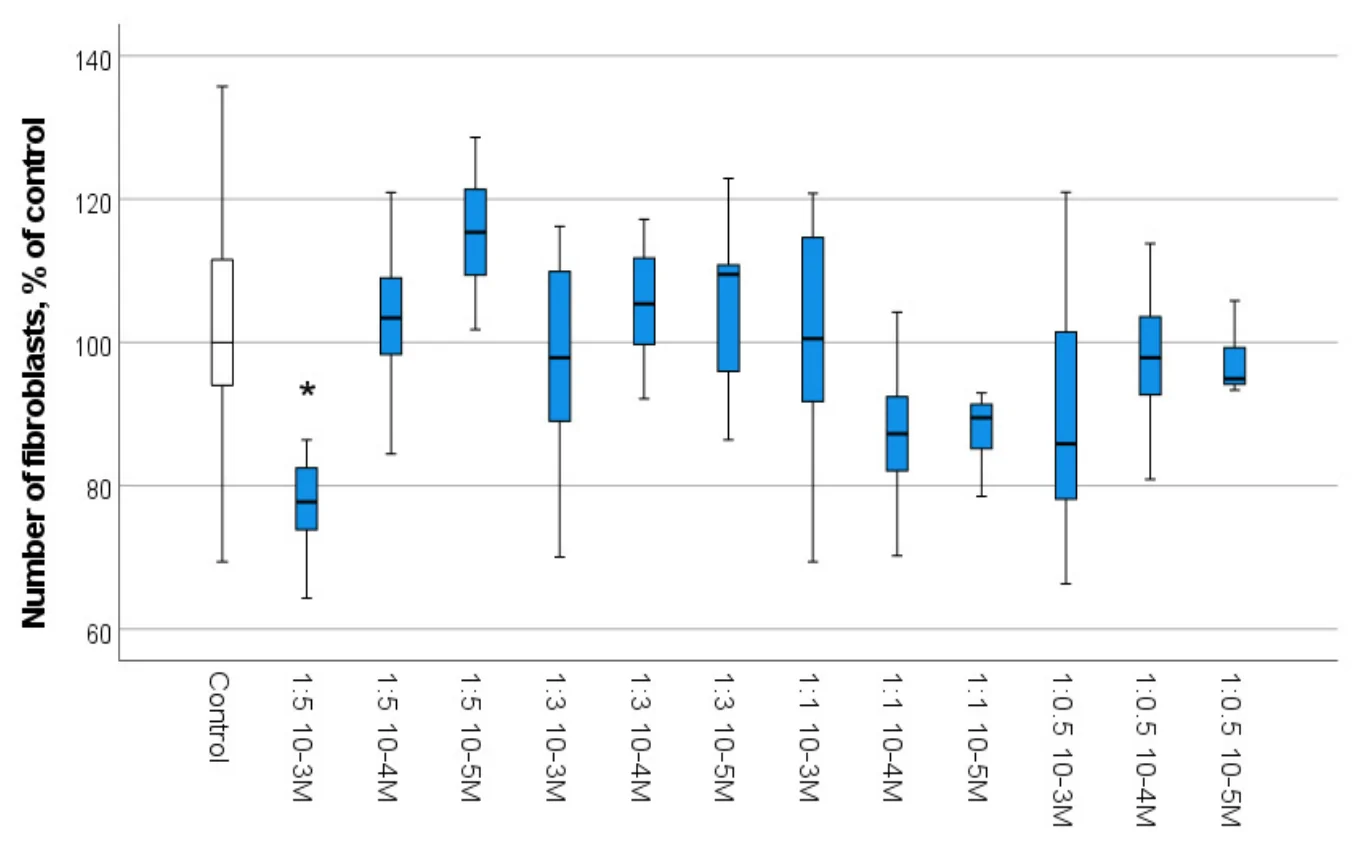
Effect of CePO_4_–CA nanocomposites with different component ratios on the proliferative activity of the human fibroblast BJ TERT cell line assessed by direct cell counting (*—*p* < 0.005 vs. control).

Evaluation of the effect of CePO_4_–CA nanocomposites on the proliferative activity of fibroblasts by direct cell counting did not demonstrate a stimulatory effect of the nanocomposites. During co-cultivation with the Ce:5CA sample at the highest concentration (10^−3^ M), inhibition of cell population proliferation by an average of 21% relative to the control was observed (Figure 9).

**Figure 10:**
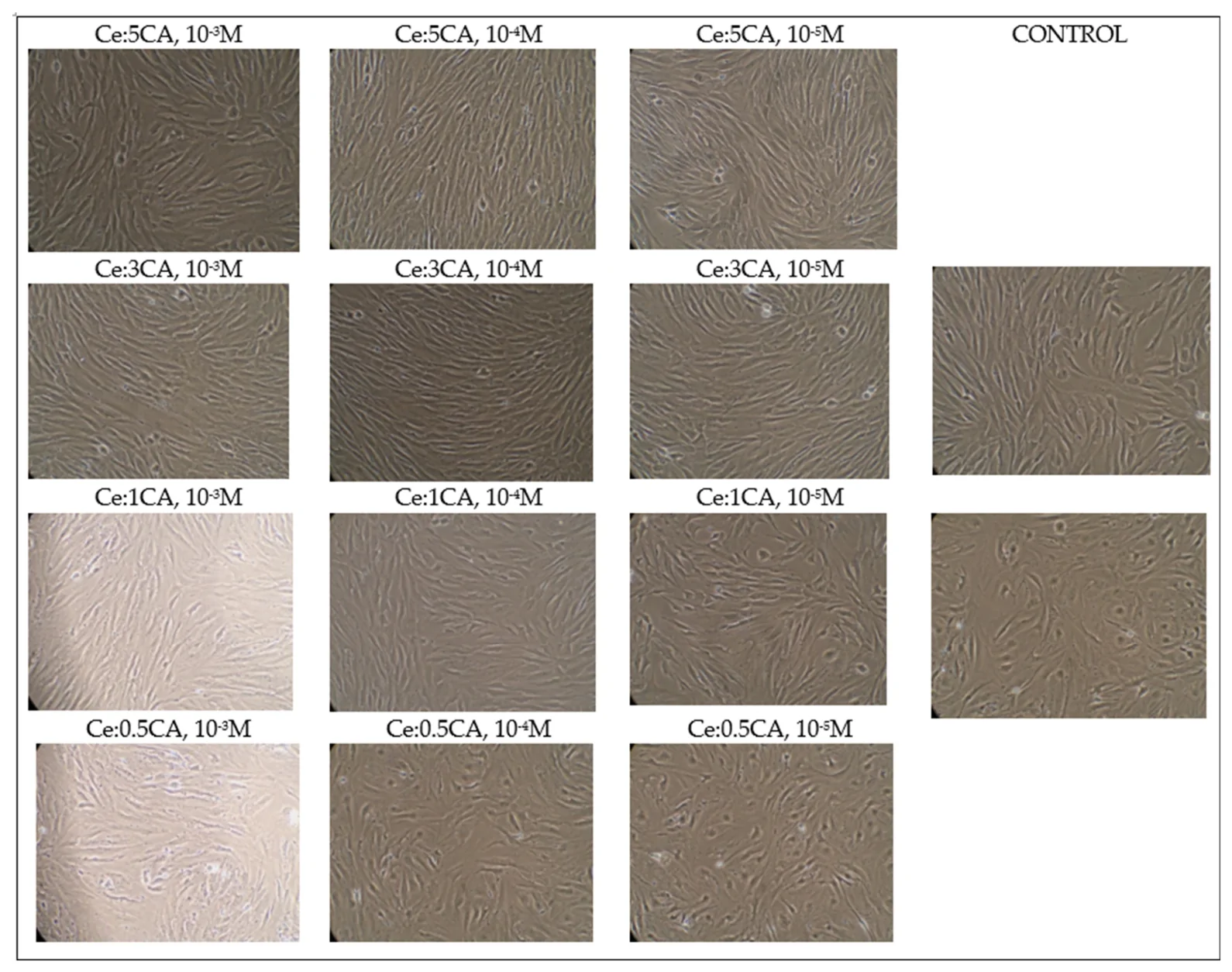
Effect of CePO_4_–Citric acid nanocomposites on the BJ TERT cell line, ×20.

A significant decrease in both parameters (OD in the MTT assay and cell number) may indicate a cytotoxic effect of the sample with the highest citrate content at a concentration of 10^−3^ M. However, it should be emphasized that no increase in the number of dead cells was detected in this sample, and the cytoarchitecture of the fibroblasts remained unchanged (Figure 10).

Overall, the obtained data demonstrate the non-cytotoxicity (biocompatibility) of the nanocomposites toward cells of the fibroblast lineage, which is fundamentally important for the development of topical cosmetic products and regenerative agents for the treatment of skin wounds. Nevertheless, since an inhibitory effect was observed at a high concentration of the Ce:5CA sample, the Ce:0.5CA sample was selected as the most promising candidate for further studies and prototype formulation development.

### 2.4. Results of Toxicological Studies in Animals

During the observation period (30 days in each experiment), no mortality was observed in any experimental group, and no external signs of toxic reactions were detected.

#### 2.4.1. Results of the Acute Oral Toxicity Study in Mice

**Figure 11:**
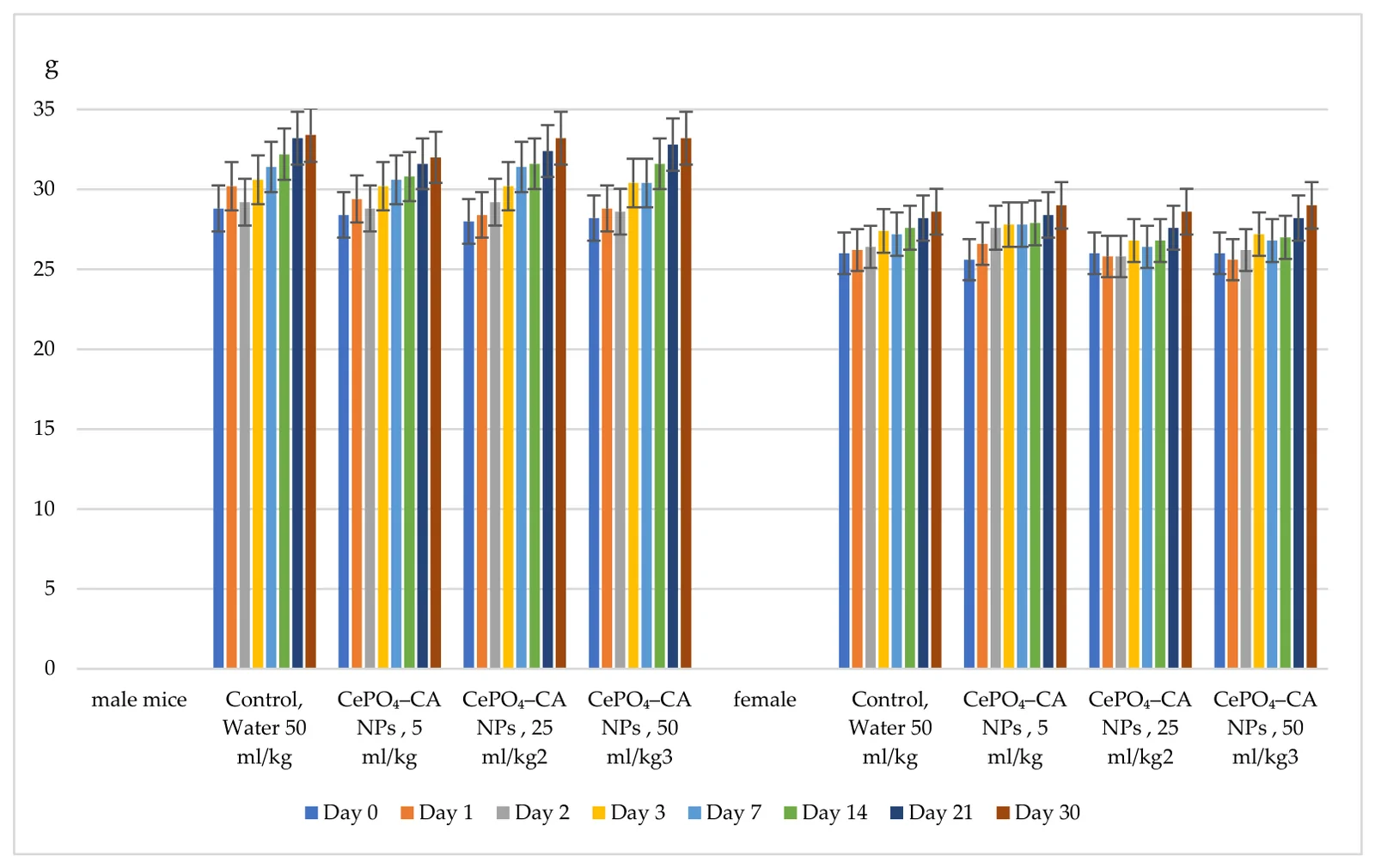
Body weight dynamics of mice during the acute oral toxicity study.

Following a single intragastric administration of the nanocomposite, all experimental animals remained alive. During the 30-day observation period, no clinical signs of toxicity were detected in any of the experimental groups. No visible disturbances of the central nervous system, autonomic nervous system, respiratory system, or excretory system were observed. The body weight dynamics of mice receiving the nanocomposite were comparable to those of the control group, with the expected increase in body weight under conditions of free access to food observed in all experimental groups (Figure 11).

Thus, the nanocomposite sol at a concentration of 10^−3^ M, following a single oral administration to CD-1 mice at doses ranging from 5 to 50 mL/kg, did not produce toxic effects and did not cause animal death. Therefore, calculation of the LD_50_ value was not possible.

#### 2.4.2. Results of the Acute Dermal Toxicity Study in Rats

In all animal groups, during the 30-day observation period following topical application of the 10^−3^ M nanocomposite at a dose of 10 mL/kg, no animals died, and no toxic reactions were observed. No visible disturbances of the central nervous system, autonomic nervous system, respiratory system, or excretory system were detected. At the site of sol application, as in the control group, no changes in skin color, rash, ulceration, or other inflammatory or degenerative changes were observed.

The body weight dynamics of rats receiving the investigated medical device prototypes during the acute dermal toxicity study were comparable to those of the control group. Under conditions of free access to food, the expected increase in body weight was observed in all experimental groups (Table 3).

Thus, the study demonstrated that CePO_4_–CA sol at a concentration of 10^−3^ M, following dermal administration at a dose of 10 mL/kg, did not produce toxic effects and did not cause animal mortality.

#### 2.4.3. Results of the Subchronic Toxicity Study of the Medical Device Prototypes in a Rat Standard Wound Model

**Figure 12:**
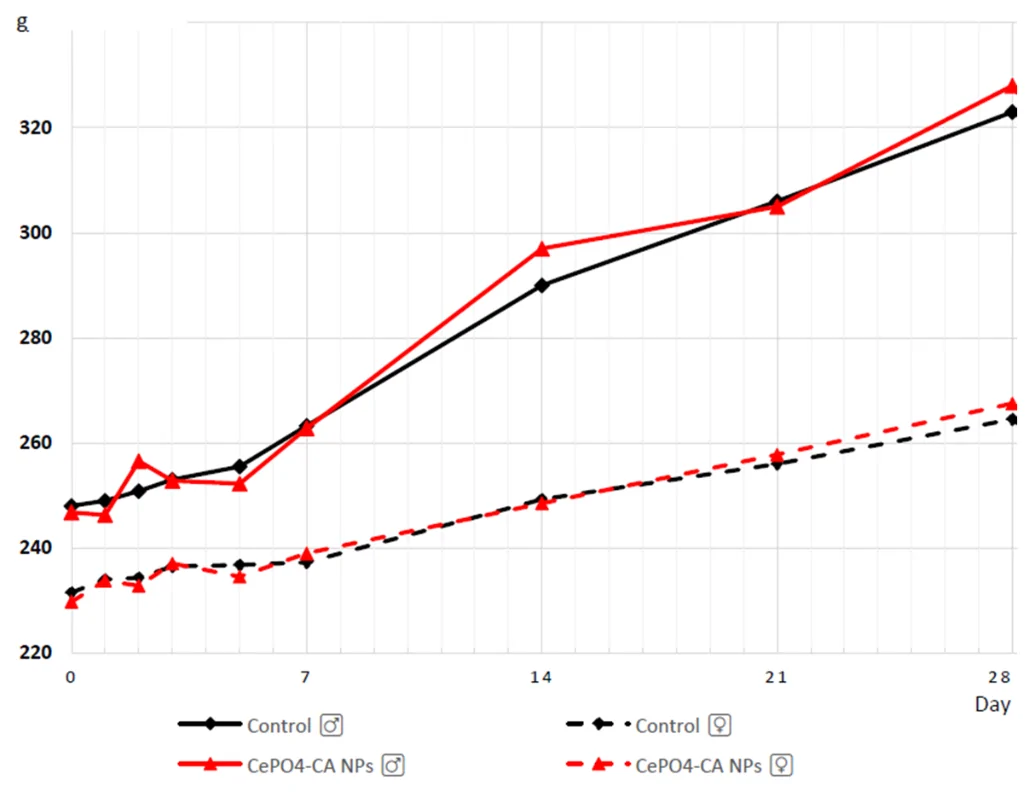
Body weight dynamics of rats during the subchronic toxicity study.

Daily application of the nanocomposite at a dose of 2 mL/kg/day to rats with experimentally induced acute full-thickness skin wounds did not affect feeding behavior or food and water consumption. Throughout the entire experimental period, the general condition, appearance, response to daily manipulations, and the appearance of both the wounds and the surrounding skin in animals treated with the nanocomposite did not differ from those of the control group. Body weight increased progressively over the experimental period in all animals, except during the first two days after wound creation, which was associated with the pain response and emotional stress following surgery. No significant intergroup differences in body weight dynamics were observed (Figure 12).

During the month treatment period by CePO_4_–CA, no significant changes in electrocardiographic parameters were detected compared with the control group (Table 4).

Blood parameters obtained from the animals on Day 30 of the experimental series remained within the reference ranges of the in-house laboratory control established for this animal species.

During gross examination of the thoracic and abdominal cavities at necropsy, no macroscopically detectable abnormalities were observed in animals of any group. The internal organs of both the control animals and the animals treated with the nanocomposite exhibited a normal gross appearance. No pathological contents were detected in the thoracic cavity, abdominal cavity, or cranial cavity.

Gross and gravimetric examination of the internal organs (liver, heart, brain, thymus, spleen, stomach, testes, ovaries, adrenal glands, small intestine, and large intestine) in rats from the control and experimental groups did not reveal any consistent pathological changes indicating systemic toxicity of the investigated nanoparticles. No statistically significant differences in the weights of the internal organs were observed compared with the control group (Table 5).

#### 2.4.4. Results of Histological Examination of Internal Organs and the Nanopreparation Application Site (Wounds) Compared with the Control

##### Microscopic Examination of the Nanodrug Prototype Application Site (Wound Area)

**Figure 13:**
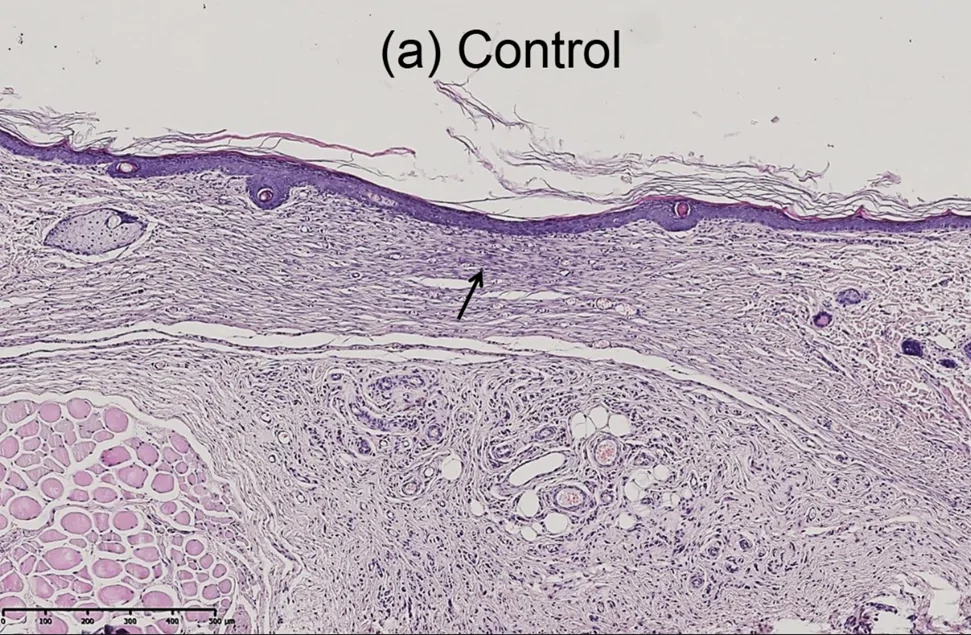

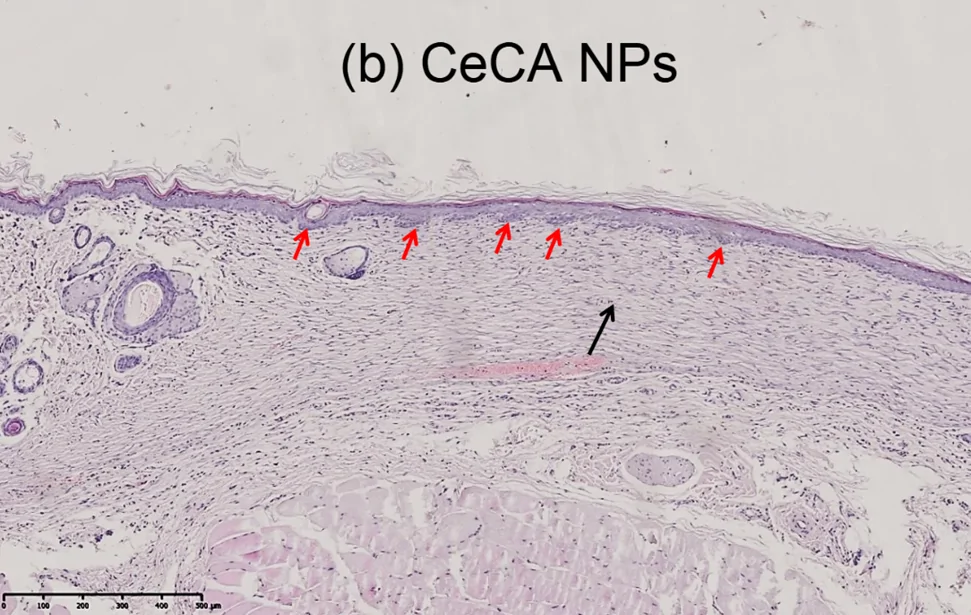
Wound area. Dense fibrous connective tissue in the wound model area (indicated by a black arrow), covered by stratified squamous keratinized epithelium (epidermis). Hematoxylin and eosin staining. Scale bar is shown in the lower left corner of the image. Epidermal appendage buds are indicated by red arrows.

Based on the histological examination of skin specimens, taking into account the presence and degree of development of dense fibrous connective tissue in the wound area, the degree of epithelialization and epidermal layer formation, the degree of basement membrane formation, the presence and degree of development of epidermal appendage buds, the degree of basophilia and spatial orientation of collagen fibers within the wound area, and the degree of papillary dermis formation, the stage of reparative regeneration in all studied groups should be characterized as completed, with transition to the scar remodeling stage. In all animals of the control group, the wound crater was completely filled with dense fibrous connective tissue composed of immature collagen fibers predominantly oriented parallel to the skin surface. In all animals, the epidermis exhibited a full-thickness structure and completely covered the scar (Figure 13a).

**Figure 14:**
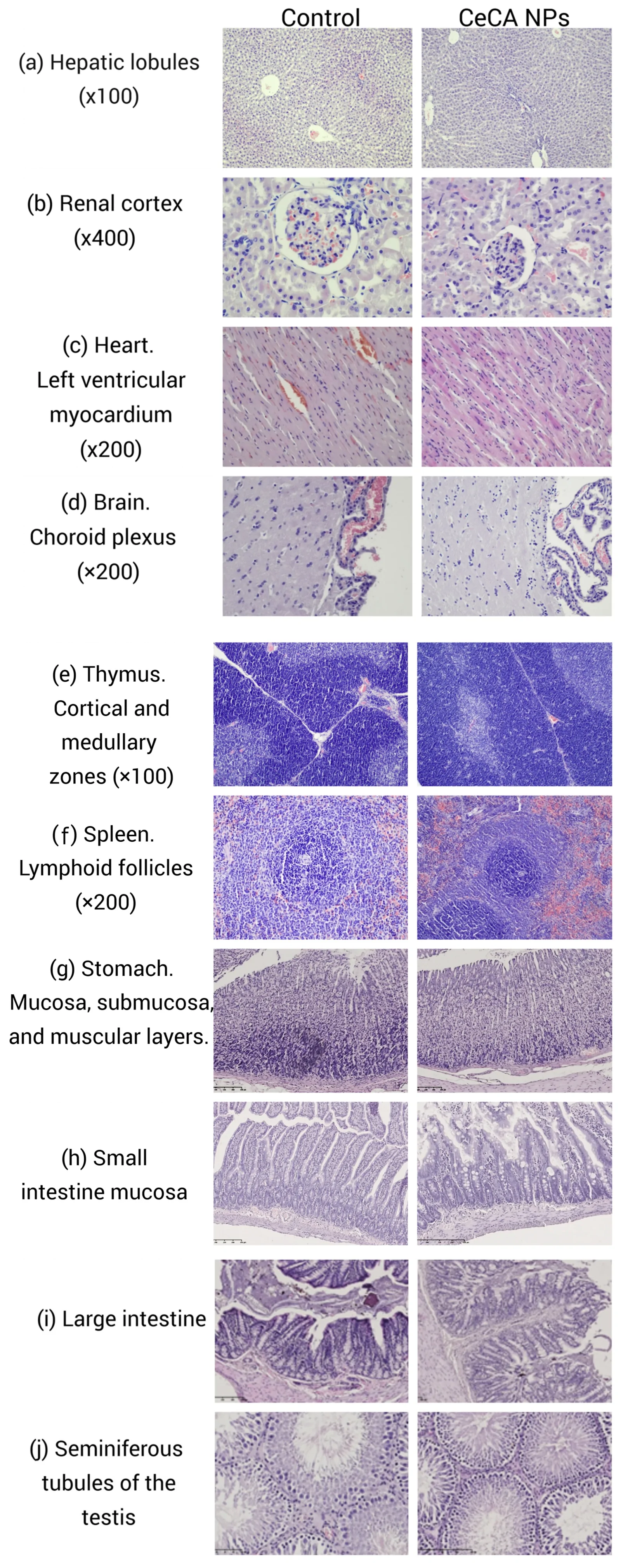
Internal organs of animals from the experimental (**right**) and control (**left**) groups. Hematoxylin and eosin staining.

In the group of animals treated with the nanocomposite, the wound crater was also completely filled with dense fibrous connective tissue. The phenomenon of “granulation tissue deficiency” was absent. It should be noted that the degree of collagen fiber maturation was higher than that observed in the control group. This was evidenced by more pronounced eosinophilia and a lower degree of organization of collagen fiber bundles. The latter indicates an earlier onset of connective tissue stromal remodeling processes in the dermis compared with the previous group. This is further supported by clusters of macrophages migrating into the deeper regions of the scar toward preserved lymphatic vessels. The epidermis was formed over the entire surface of the dense fibrous connective tissue and was separated from it by a basement membrane. It exhibited a five-layer organization throughout the entire surface. In 50% of animals, well-formed epidermal appendage buds were observed, predominantly localized at the edge of the wound crater (Figure 14b).

##### Microscopic Examination of Internal Organs

In animals treated with CePO_4_–CA NPs at a concentration of 10^−3^ M, the histological structure of liver tissue corresponded to that of the control group. Hepatic lobules were properly formed, consisting of radially oriented and well-defined cords of hepatocytes. Hepatic sinusoids were moderately congested. Hepatocytes were morphologically uniform, with no signs of dystrophic or necrotic changes (Figure 14a).

The histological appearance of the kidneys also did not differ from that of the control rats. The structural organization of the glomerular apparatus was preserved, and no destructive or inflammatory changes were observed in the glomeruli. Renal convoluted tubules showed normal histological structure, and the epithelial lining exhibited no dystrophic or necrotic changes. The walls of the renal pelvis showed no signs of inflammation, and the epithelial lining was preserved (Figure 14b).

In both groups of animals, the myocardium of both ventricles exhibited normal structure and consisted of three layers of orderly arranged cardiomyocytes without signs of dystrophic or necrotic changes. No inflammatory or other pathological alterations were observed in the interstitial tissue, endocardium, or pericardium (Figure 14c).

The morphological structure of the brain tissue did not differ between the experimental and control groups. The pia mater was thin and free of inflammatory changes, with thin-walled vessels showing moderate and uneven blood filling. The cerebral cortex demonstrated a distinct layered organization, while glial cells and neurons showed no signs of cell death. No evidence of brain edema was observed. The choroid plexuses of the ventricles were well developed and showed no pathological changes (Figure 14d).

The thymus also showed no morphological differences. The lobular architecture was preserved, and corticomedullary differentiation was distinct. The cortical zone consisted of densely packed small lymphocytes without signs of delymphatization. In the medullary zone, single clusters of thymic epithelial cells were observed, while Hassall’s corpuscles were not detected (Figure 14e).

The spleen exhibited a uniform histological structure corresponding to normal rat morphology. Lymphoid follicles were well defined, of normal size, mostly without germinal centers, with wide mantle zones and clearly delineated boundaries. Periarteriolar lymphoid sheaths were densely populated with lymphocytes. No abnormalities in cellular composition or histoarchitectonics were observed (Figure 14f).

The gastric mucosa showed no differences between groups. The cardiac region was covered by stratified squamous keratinized epithelium with a thin surface keratin layer. Gastric glands, consisting of neck, body, and base regions, contained all types of exocrine cells (chief, parietal, and mucous cells) with typical distribution. No degenerative, inflammatory, or other pathological changes were observed in the mucosa, submucosa, muscular layer, or serosa of the stomach (Figure 14g).

The small intestine showed no morphological differences between the two groups. Villi were well defined, and their epithelium showed no destructive changes. The cellular composition of the villous stroma was identical to that of the control group. No inflammatory changes were observed in the mucosa, submucosa, or muscular layer of the intestinal wall (Figure 14h).

No histoarchitectural alterations were observed in the large intestine. In both groups, the mucosa exhibited normal structure, and the crypt epithelium showed no degenerative or inflammatory changes in the stroma, submucosa, or muscular layer (Figure 14i).

The reproductive system also showed no abnormalities. In males, mainly cross-sectioned seminiferous tubules were observed beneath the tunica albuginea of the testes. The seminiferous epithelium contained 2–4 generations of germ cells depending on the stage of spermatogenesis. No pathological changes were detected in the germinal epithelium, spermatozoa, or testicular connective tissue stroma (Figure 14j). In females, no pathological changes were observed in the ovarian cortex. The cortical substance was predominantly occupied by corpora lutea remaining from previous cycles. Secondary follicles of different maturation stages, as well as tertiary follicles, did not differ between the studied groups. No pathological changes in the vascular bed or tubular structures were observed in the ovarian medulla or hilum.

Thus, the histological and gravimetric examination of the internal organs and wounds of animals in both experimental and control groups did not reveal any consistent pathomorphological changes indicative of systemic toxic effects of the investigated preparations on the animal body.

## 3. Discussion

There is a growing interest in the development of drugs based on nanoparticles of rare earth elements, which is associated with the discovery of beneficial biomedical effects such as antioxidant, antibacterial, and regenerative properties, most extensively studied for cerium oxide nanoparticles [1,2,3,4,5,6,7,8,54,55,56].

Cerium phosphate is a promising candidate for the development of a new generation of highly effective regenerative agents. It possesses many properties similar to cerium dioxide (such as antioxidant and regenerative activity), as well as specific characteristics inherent to its phosphate form [22,26,27,28,49,50,51,57,58,59]. The conducted studies have shown that successful synthesis and biomedical application of cerium phosphate nanocompounds require a number of mandatory conditions, without which the intended bioactive effect cannot be achieved [22,23,24,25,26,27,28]. Many of these conditions are not typical for routine pharmacology. It is possible that these circumstances hinder the translation of cerium-based nanocompounds into practical applications. Within the scope of this article, the authors have attempted to discuss some of these aspects.

Due to the high aggregative tendency of cerium-based nanoparticles, the choice of a stabilizing agent preventing aggregation of the synthesized compounds is of particular importance. The antioxidant properties of citric acid are well known and widely applied in the food, cosmetic, and pharmaceutical industries [36,37,38,39,40,41,42,43]. Its antimicrobial activity has been studied against a range of bacteria (*E. coli*, *S. aureus*, *C. albicans*, *Listeria monocytogenes*, *Salmonella typhimurium*, *Vibrio parahaemolyticus*) by various authors [36,60,61]. There are also data on the bacteriostatic effect of CeO_2_ nanoparticles coated with a citrate shell [38]. Therefore, the use of citrate as a stabilizer for cerium orthophosphate nanoparticles appears promising from both chemical and biological perspectives.

This study is a continuation of our series of works on the synthesis and research of nanosized cerium phosphate. In our previous works, we identified the optimal conditions for obtaining precipitates with the minimum particle size: temperature, precipitation time, pH of the solutions, and the order of their mixing [21,22,49]. The task of minimizing particle size was set based on the dimensions of cellular structures in living organisms, to enable their optimal use in biomedical applications. At the same time, there is evidence [52] of the manifestation of antioxidant properties by cerium particles of significantly larger sizes (more than 70 nm).

These conditions were used to obtain shell-like cerium nanophosphates. It is worth noting that this study focuses on the ratio of cerium component and citric acid, which will also be taken into account in subsequent research.

Cerium phosphate nanocomposites stabilized with citrate at different ratios, obtained by chemical precipitation using ammonium dihydrogen phosphate from a nitrate solution, were characterized by physicochemical methods, first to confirm the size and morphology of the synthesized crystals, and second to provide objective evidence of the formation of a new material. XRD and TEM analyses demonstrated their single-phase nature and fine dispersion; in all cases, the structure of cerium orthophosphate corresponded to the rhabdophane phase. A slight distortion of the structure was observed in the non-annealed samples, which decreases after annealing (200 °C, 2 h), which appears entirely logical. According to TEM data, the morphology of nanoparticles for all studied Ce:CA c ratios (1:5–1:0.5) was characterized by anisotropic nanocrystals (nanorods) with a bimodal size distribution, with widths of approximately 3–10 nm and lengths of 15–40 nm. Among all samples, a tendency toward lower aggregation and more pronounced visualization of atomic lattice planes was observed in Ce:1–0.5CA samples. It can be assumed that the optimal ratio of initial reagents is cerium: citrate 1:0.5–1:1.

It was established that citric acid, introduced into the reaction medium as a stabilizing agent, affects the morphology of the obtained precipitates by limiting the linear growth of crystals compared to those obtained by us in media. Similar results regarding the effect of citric acid on the dimensional characteristics of rare-earth metal phosphate particles have been reported in other studies [21,33,37,38,42,45].

The performed IR spectroscopy of the obtained samples allowed the following to be determined. First, in all non-calcined samples, absorption bands corresponding to P–O bond vibrations were detected, as well as a shift of carbonyl group absorption bands in the 1800–1600 cm^−1^ region, indicating the formation of new bonds affecting the vibrations of the carbonyl group itself. A shift in absorption bands for skeletal –C–C– vibrations of citric acid was also observed, confirming the formation of a nanocomposite material, i.e., effectively a new nanomaterial with new properties. Second, calcination of the samples for 2 h at 200 °C (conditions previously used for obtaining nanosized cerium phosphates) leads to gradual degradation of the nanocomposite with respect to its citrate shell. In the case of a high excess of citric acid, citric acid appears to decompose first, followed by degradation of the nanocomposite itself, as seen from the curve of the Ce:5CA sample. In contrast, at low excess (1:0.5 ratio), almost all characteristic absorption bands of citric acid disappear, leaving only the bands characteristic of cerium phosphate. When the outer shell is lost due to thermal treatment, an increase in aggregation properties and a decrease in antioxidant activity of the nanocomposite should be expected.

Thus, IR spectroscopy confirms the presence and coordination of citric acid in non-calcined samples. Thermal treatment (200 °C, 2 h) leads to partial or complete removal of citric acid depending on its initial excess. This justifies the advantages of non-calcined nanocomposites of the CePO_4_–CA system and supports the choice of a synthesis route without thermal treatment for subsequent studies aimed at drug development.

Oxidative stress-modulating inorganic nanoparticle-based nanomaterials have promising advantages in nanomedicine due to their ability to provide a stable and reliable method of delivering a biochemical complex of interrelated components, including free radicals, proteins, enzymes, metabolites [1,3,62,63]. The redox activity of cerium nanoparticles, the most studied rare earth element with variable valence and unique physical and chemical properties, has inspired the scientific community to develop various technical and biomedical applications [5,6,7,8,64,65,66,67,68].

To study redox activity, we used the chemiluminescence method. Chemiluminescence-based approaches occupy an important place among methods for assessing redox activity because of their high sensitivity, rapid response, and ability to detect changes in redox processes in real time without an external excitation source [69,70]. The use of modified chemiluminescent systems for the analysis of nanomaterials is particularly promising, since such models make it possible to simultaneously evaluate the anticipated antioxidant and pro-oxidant properties of samples and correlate them with biologically relevant oxidative processes [71,72].

Redox-activity studies demonstrated that the antioxidant properties of citrate-stabilized cerium phosphate nanoparticles strongly depend both on the ratio of stabilizer (citric acid, in this case) to cerium and on the concentration of nanosols in a methionine-modified Fenton reaction model system. In particular, increasing the concentration of Ce:3CA, Ce:1CA, and Ce:0.5CA composites from 10^−5^ to 10^−3^ M resulted in a pronounced increase in antioxidant capacity, reaching maximum values at 10^−3^ M. At the same time, the antioxidant activity significantly exceeded that of ascorbic acid at the same 10^−3^ M concentration by 1.5–2 times. This beneficial antioxidant effect is consistent with other studies [44,46,47,48,49,50,52,62] and effect may be useful in various diseases and pathological conditions, as well as in regenerative medicine for the treatment of acute and chronic wounds, and in aesthetic and restorative cosmetology. In contrast, the Ce:5CA sample exhibits predominantly pro-oxidant properties across all studied concentrations, most strongly expressed at the highest concentration of 10^−3^ M, which may potentially be used, for example, in theragnostic.

The obtained data emphasize dose-dependent redox activity and are consistent with the dual role of cerium-containing nanomaterials described in the literature, which can act as both antioxidants and pro-oxidants depending on conditions and nanoparticle surface composition. In particular, nanoscale double ceric phosphates were reported to act as antioxidants toward alkylperoxyl radicals and prooxidants toward hydrogen peroxide [52]. Amorphous CePO_4_ showed better low-temperature redox performance in SCR catalysis, while crystalline CePO_4_ had poorer redox properties but stronger acid-site behavior [73]. In a CePO_4_/CeO_2_ nanocomposite, Ce^3+^ from CePO_4_ promoted ozone activation to hydroxyl radicals, showing catalytic redox participation [74]. At the same time, there is evidence that the formation of cerium phosphate blocks Ce^3+^/Ce^4+^ cycling, reducing superoxide dismutase/catalytic antioxidant activity in cerium oxide systems [48].

We believe that the high concentration of citric acid is the factor that determines the prooxidant activity. We hypothesize that at low or moderate Ce:CA ratios, citrate forms a thin layer on the surface of cerium phosphate, maintaining access to the surface active sites of Ce^3+^/Ce^4+^, where SOD (Superoxide Dismutase) and catalase-like reactions of neutralization of reactive oxygen species occur. With a large excess of citric acid, its molecules form a continuous dense layer, thereby shielding the surface cerium ions and depriving them of the ability to catalytically decompose hydrogen peroxide. This releases free citrate into solution, where it acts as a prooxidant catalyst.

In cell experiments on human fibroblasts, a significant decrease in all indicators (OD in the MTT assay and cell number) was observed upon co-cultivation with Ce:5CA at 10^−3^ M, which may indicate a cytotoxic effect of this sample.

Overall, the remaining nanocomposites demonstrate biocompatibility toward the fibroblast lineage, which is consistent with previously reported studies and is critically important for the development of topical cosmetic products and regenerative agents for skin wound healing.

Biocompatibility and non-cytotoxicity of cerium phosphate nanoparticles have also been demonstrated in the works of other researchers. Amorphous and crystalline cerium phosphates did not show toxicity to mouse fibroblasts or human mesenchymal stem cells, and enhanced MSC proliferation [50]. CePO_4_-based nanomaterials were described as biocompatible across a broad concentration range in HaCaT skin cells, with minimal photocatalytic activity and photoprotective behavior [75]. Therefore, the safety of CePO_4_-based nanomaterials makes them very attractive for biomedical and cosmetic purposes.

Due to effectiveness across a wider concentration range and its higher antioxidant capacity compared to ascorbic acid, the Ce:0.5CA sample was selected for further studies, as it demonstrated the best stimulatory effect on human fibroblasts during co-cultivation experiments.

Based on the toxicological studies of the selected nanocomposite Ce:0.5CA in sol form at a concentration of 10^−3^ M, it can be concluded that the nanocomposite does not exert any noticeable systemic toxic effect on the animal organism at the studied doses. Acute toxicity studies in mice after a single oral administration of the nanosol at doses from 5 to 50 mL/kg (up to 11.75 mg/kg in terms of CePO_4_), as well as dermal application to intact shaved dorsal skin of rats at a dose of 10 mL/kg (2.35 mg/kg in terms of CePO_4_), revealed no toxic effects and no mortality throughout the entire observation period. In the subchronic toxicity study in rats with a model of standardized full-thickness square skin wounds on the back, followed by daily application of the nanocomposite at a concentration of 10^−3^ M first to the wound and surrounding tissues and, during wound healing, to regenerating skin for one month at a dose of 2 mL/kg (0.47 mg/kg in terms of CePO_4_), no toxicity or mortality was observed. Body weight dynamics, feed and water intake, and the general condition of animals in the treated groups did not differ from the control group throughout the experiment. No significant adverse effects on hematological and biochemical blood parameters or internal organs were detected. No evidence of irritant effects on intact skin, wounds, surrounding tissues, or regenerating skin was observed throughout the study. The obtained data on the non-toxicity of cerium-containing nanoparticles for mammals are consistent with numerous studies summarized in systematic review devoted mainly to CeO_2_ nanoparticles [65]. There are currently no comprehensive data on the safety of using CePO_4_ nanoparticles in vivo, which adds interest to our work.

The results of our study provide a basis for further in vivo studies aimed at evaluating the efficacy of treating acute and infected skin wounds. These will be the focus of our future research.

## 4. Materials and Methods

### 4.1. Synthesis of Nanocomposites Using Citric Acid and Cerium Nitrate at Different Ratios

The synthesis was carried out using the following reagents: cerium nitrate hexahydrate, Ce(NO_3_)_3_·6H_2_O (99.99%, LANHIT, Moscow, Russia; analysis of the impurity composition of the initial cerium nitrate was performed at the Shared Research Center of the Kurnakov Institute of General and Inorganic Chemistry, Russian Academy of Sciences); anhydrous citric acid, C_6_H_8_O_7_ (99.5%, Ruskhim, Moscow, Russia); and ammonium dihydrogen phosphate, NH_4_H_2_PO_4_ (98%, Ruskhim, Moscow, Russia).

**Figure 15:**
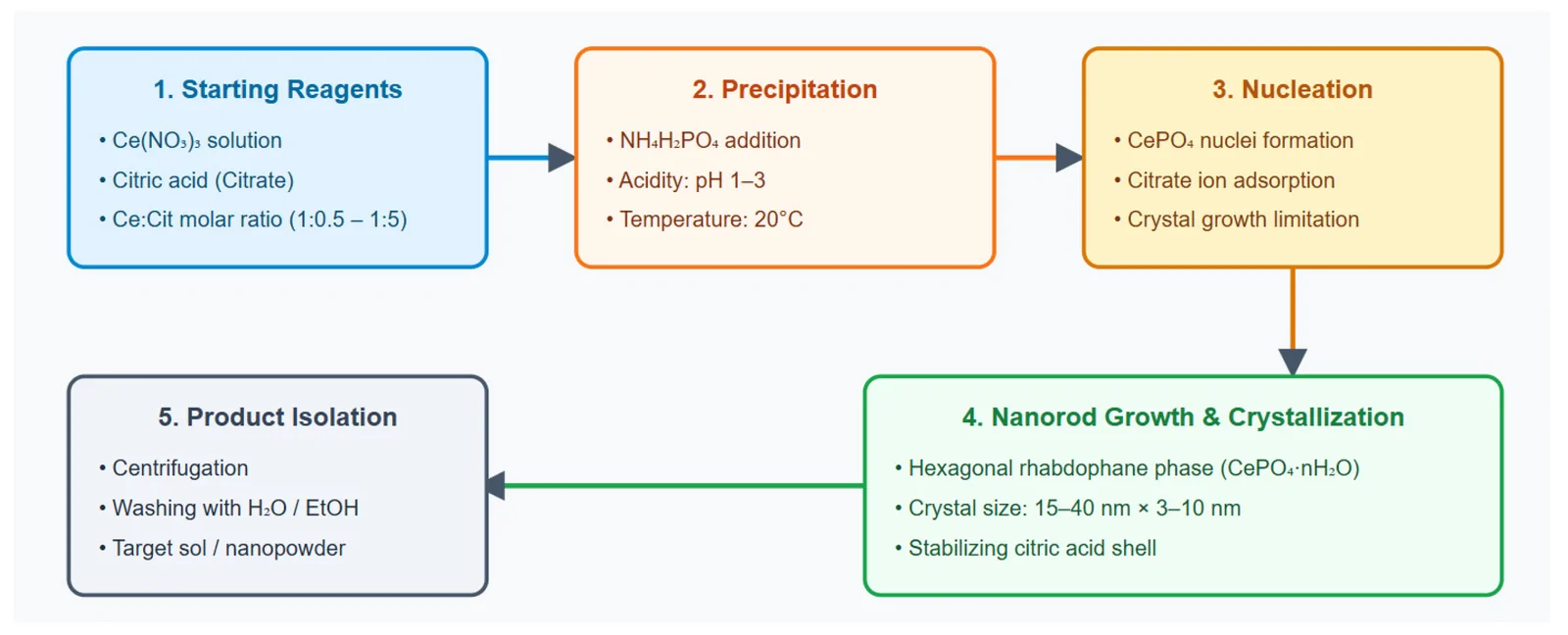
Preparation scheme of citrate-stabilized CePO_4_ nH_2_O nanoparticles.

A cerium nitrate solution was prepared with an initial concentration of 125 g/L, calculated as CeO_2_. The composition of the initial solution was then varied by adding the stabilizer at a predetermined concentration (Table 6, Figure 15).

In general, the synthesis was carried out as follows. Solutions of Ce(NO_3_)_3_ and citric acid (within a mass ratio range of 1:5 to 1:0.5) were placed into a reaction vessel under continuous stirring. A solution of NH_4_H_2_PO_4_ with a concentration of 100 g/L was then added by spraying. The cerium phosphate precipitate was allowed to stand for 12 h, after which it was filtered and dried at room temperature to a residual moisture content of approximately 2%. To evaluate changes in the physicochemical properties of the powders at the limiting citric acid contents, the Ce:5CA and Ce:0.5CA samples were heat-treated at 200 °C for 2 h.

### 4.2. Methods for Evaluating the Physicochemical Properties of the Nanocomposites

The structural and morphological features of the nanoparticles were studied by transmission electron microscopy (TEM) using a high-resolution JEOL JEM 2100 microscope (JEOL, Akishima, Tokyo, Japan) at the Collective Use Center “Materials Science and Metallurgy” of the National University of Science and Technology MISIS. A LaB_6_ cathode was used as the electron source. The powder structure was examined by transmission electron microscopy at an accelerating voltage of 200 kV. A 40 μm objective aperture was used for high-resolution imaging. A condenser aperture with a diameter of 50 μm was used to ensure spatial coherence. The average electron beam diameter during image acquisition was approximately 100 nm (3 Spotsize). Images were acquired 2 h after the accelerating voltage of 200 kV had been switched on to allow thermal stabilization of the microscope column. The accelerating voltage stability was 2 × 10^−6^ min^−1^. The objective lens current stability was 1 × 10^−6^ min^−1^. TEM examination was performed first for the powders and then for the sols with a CePO_4_ concentration of 10^−4^ M.

The nanoparticle size distribution was determined by visual counting of a sufficient number of particles (at least 100) of similar size within each selected size category. The counting was performed in several regions of the TEM micrograph obtained for each sample.

The phase composition of the CePO_4_–CA nanocomposites was determined by X-ray diffraction using a Bruker D8 diffractometer (Bruker AXS, Karlsruhe, Germany) equipped with a Bruker scintillation detector. The study was carried out in the Collective Use Center “Materials Science and Metallurgy” of the National University of Science & Technology MISIS. The phase composition and the size of the coherent scattering domains of the samples were determined using the θ–2θ scanning geometry. Monochromated CuKα radiation (λ = 0.154 nm) was used. The diffractometer operating conditions were a voltage of 40 KV and a tube current of 40 mA.

The composition of the synthesized composites was analyzed by infrared spectroscopy. The measurements were performed using a Bruker VERTEX 70 Fourier-transform infrared spectrometer (Bruker, Karlsruhe, Germany) equipped with a PIKE MIRacle attenuated total reflection (ATR) accessory with a diamond crystal on a zinc selenide prism. The spectra were recorded under the following conditions: 32 scans; spectral resolution of 4 cm^−1^; spectral range of 4000–600 cm^−1^. The background spectrum was recorded immediately before each measurement.

The cerium content in the sample sols was determined by inductively coupled plasma mass spectrometry (ICP-MS) using an Elan DRC-e instrument (PerkinElmer, Madrid, Spain). Calibration was performed using a multi-element calibration standard for ICP-MS (MS68-AB, 100 μg/mL; High-Purity Standards, North Charleston, SC, USA), serially diluted to 20 ppb. A 2% nitric acid solution served as the blank. Diffraction peaks were identified using data from the Joint Committee on Powder Diffraction Standards (JCPDS, Swarthmore, PA, USA).

### 4.3. Methods for Assessing the Redox Activity of CePO_4_–CA Nanocomposites

Redox activity was measured using a Lum-100 instrument (DISoft, Moscow, Russia) with PowerGraph 3.3.12 software. A methionine-modified Fenton reaction was used as the basis of the chemiluminescent assay.

For the control experiment, 100 μL of iron(II) sulfate heptahydrate solution (pure grade, RuskhimI, Kaluga, Russia) at a concentration of 10^−3^ M (calculated as Fe^2+^), acidified with sulfuric acid (chemically pure grade, Sigma Tek, Khimki, Russia) to pH = 2, was added to the chemiluminometer tube. Subsequently, 100 μL of L-methionine solution (USP grade, PanEco, Moscow, Russia) at a concentration of 1.6 × 10^−5^ M was added, and the aliquot volume was adjusted to 2.4 mL with deionized water acidified with sulfuric acid to pH 2. To initiate chemiluminescence, 100 μL of a 3% hydrogen peroxide solution (Ekoteks, Moscow, Russia), diluted with deionized water to a concentration of 3 × 10^−3^ M, was added, after which the signal was recorded at 37 °C.

To evaluate the antioxidant activity of the samples at different concentrations, the corresponding suspensions were added in a volume of 100 μL before the addition of hydrogen peroxide, giving a total sample volume of 2.5 mL. Ascorbic acid (chemically pure grade, Areolab, Saint-Petersburg, Russia) at the same concentrations as the CePO_4_–CA nanocomposites served as the reference antioxidant. Luminescence recording was initiated automatically, and the interval between the start of the reaction and the beginning of signal acquisition did not exceed 1 s. Chemiluminescence was recorded in relative units of PPS (pulses per second). The acquisition frequency was 1 Hz. At least five measurements were performed for each group at each concentration (*n* = 5).

For the control methionine-modified Fenton reaction, chemiluminescence was recorded for 10 min. After 110 s from the start of the reaction, a plateau was observed on the kinetic curve, corresponding to completion of the reaction. The area under the curve, representing the integral light sum (S), was then calculated for each experiment using PowerGraph 3.3.12 software over a period of 120 s from the start of the reaction until complete cessation of chemiluminescence.

### 4.4. Biomedical Studies Using Cell Line In Vitro

The experiments were performed on BJ TERT cell line (a modified line of primary human fibroblasts, American Typed Cell Collection, Manassas, VA, USA). Cell culture was performed in Petri dishes (SPL Life Sciences, Pocheon-si, Republic of Korea) using a standard protocol in complete DMEM medium with high glucose (PanEco, Russia). Cell cultures were maintained in a CO_2_ incubator (Binder, Tuttlingen, Germany) under standard controlled conditions (5% CO_2_, 37 °C) and controlled humidity.

Biocompatibility, cytotoxicity, and the effects of nanocomposite sols over a wide concentration range (10^−3^ M–10^−5^ M) on metabolic and proliferative activity were evaluated using the MTT assay and direct cell counting with determination of the dead/live cell ratio after 72 h of co-culture with the nanocomposites, according to proven and standardized methods, described in detail earlier [22,49]. The MTT assay was performed using a Multiscan Labsystems spectrophotometer (Vantaa, Finland) at a wavelength of 540 nm. Results were expressed as relative optical density (OD). Cell counts were determined automatically using a Countess II Automated Cell Counter (Thermo Scientific, Waltham, MA, USA). The study was performed with 12 replicates (*n* = 12).

In the biomedical study, concentration (10^−3^ M, 10^−4^ M, 10^−5^ M) means the concentration of the initial nanocomposite sol added to the well in a volume of 10%. Distilled water added to the wells in the same volume as the nanocomposites (10% *v*/*v*) served as the control.

### 4.5. Toxicological Studies with Safety Evaluation in Different Animal Species

The animals (mice and rats) were obtained from the Stolbovaya Branch of the Scientific Center for Biomedical Technologies of the Federal Medical Biological Agency. After receipt, quarantine, and an acclimatization period of at least 10 days, the animals were randomly assigned to the experimental groups according to body weight (the average weight of the animals in the group), and the studies were performed. The total number of animals used—68 (40 CD-1 mice and 28 Wistar rats). The animals were kept under standard vivarium conditions with a 12-h light/12-h dark cycle and free access to water and food.

To comply with the commonly accepted ‘3Rs’ (https://nc3rs.org.uk/who-we-are/3rs, accessed on 5 August 2026) principles, in particular “Reduction in number of animals used”, it was decided to use the minimum number of study units with the approval of the ethics committee. It was planned that if an animal died earlier, an additional animal would take its place. But there was no premature death of animals.

Toxicological studies were carried out using a sol of the selected CePO_4_-0.5CA nanocomposite at a concentration of 10^−3^ M.

#### 4.5.1. Ethics

The study was approved by two ethics committees. The study protocol was approved by the Local Ethics Committee of Sechenov University (Protocol No. 19–23, 26 October 2023) and by the Regional Ethics Committee of Kursk State Medical University, Ministry of Health of the Russian Federation (Protocol No. 1, 4 March 2024).

#### 4.5.2. Acute Oral Toxicity Study in Mice

Acute toxicity following a single oral administration of CePO_4_–CA nanoparticles NPs was evaluated in 40 CD-1 white laboratory mice of both sexes using a metal gavage needle (0.8 mm in diameter, with a smooth olive-shaped tip) to assess the potential systemic effects of the nanocomposite. The body weight of the mice before the start of the study ranged from 24 to 30 g.

Since the LD_50_ value had not previously been determined for the investigated cerium phosphate-based medical device prototypes, the highest dose corresponded to the maximum permissible volume for a single oral administration to mice 50 mL/kg. The lowest dose was selected as 10% of the maximum dose (5 mL/kg), and the intermediate dose was 25 mL/kg. Dilutions were prepared using water for injection. Animals in the control group received a single oral administration of water for injection at a dose of 50 mL/kg.

After the quarantine period, the animals were randomized into four subgroups of 10 animals each (5 males and 5 females): water for injection (control group, 50 mL/kg) and CePO_4_–CA (10^−3^ M) administered at doses of 50, 25, or 5 mL/kg.

The toxic effects of the medical device prototypes were assessed based on the clinical condition of the animals and their survival (LD_50_) over a 30-day observation period.

#### 4.5.3. Acute Dermal Toxicity Study in Rats

Acute dermal toxicity of the nanocomposite was evaluated in 12 Wistar rats of both sexes (male body weight: 260–320 g; female body weight: 220–260 g) following application to intact skin. The animals were divided into two experimental groups, each consisting of six animals (three males and three females).

Under isoflurane anesthesia, the hair on the dorsal surface of each rat was shaved from the level of the scapulae caudally in a strip approximately 3 cm wide and 5–6 cm long. The prototype nanomedicine was applied once and evenly to the depilated skin area (15–18 cm^2^). The maximum volume of the medical device that did not spread beyond the marked skin area was determined empirically to be 0.2 mL per 1.5 cm^2^ of skin. The maximum dose of the nanocomposite was 10 mL/kg body weight (2.35 mg/kg calculated as cerium phosphate). Animals in the control group received water for injection applied to the skin at the same dose (10 mL/kg).

The toxic effects of the CePO_4_–CA nanocomposite were evaluated based on the clinical condition of the animals and their survival over a 30-day observation period.

#### 4.5.4. Subchronic Toxicity Study of the Medical Device on a Standard Rat Wound Model

Subchronic toxicity of the CePO_4_:0.5CA sol at a concentration of 10^−3^ M was evaluated in 16 Wistar rats of both sexes. Since the intended clinical application of the developed medical device involves application to the wound and the periwound area, the subchronic toxicity study was performed using a standard wound model. The animals were randomly assigned to two experimental groups. Each group consisted of eight animals (four males and four females). Before the start of the study, the body weight of the males ranged from 225 to 270 g, and that of the females from 220 to 250 g.

Under isoflurane anesthesia, two standardized wounds were created on the back of each rat at equal distances to the right and left of the spine (wound size: 1 × 1 cm; depth: to the fascia). The skin incision was made using sterile Beroblade №15 surgical blades (Beromed GmbH Hospital Products, Berlin, Germany).

The dose of the test preparation was selected based on the initial wound size and the results of the preliminary acute toxicity studies and amounted to 0.2 mL per wound. The total dose administered to each rat immediately after wound creation was 0.4 mL per rat weighing approximately 200 g, or 2 mL/kg (0.47 mg/kg calculated as cerium phosphate). Throughout the subchronic toxicity study, the test sol was applied to the wound area once daily at a dose of 2 mL/kg regardless of changes in wound size. Animals in the control group received water for injection applied to the wound area once daily at the same dose (2 mL/kg).

Each animal underwent a daily visual clinical examination for 30 days from the start of the experiment. During the examination, attention was paid to the general condition of the animals, locomotor activity, respiratory rate, the condition of the skin, visible mucous membranes, and wound surfaces.

Body weight was measured using an A&D-5001S balance (A&D, Tokyo, Japan) on Day 0 before wound creation, on Days 1, 2, 3, and 7, and every 7 days thereafter.

Immediately before wound creation and every 7 days thereafter, electrocardiography (ECG) was recorded in three standard leads using a Mortara Eli150L electrocardiograph (Mortara Instrument, Inc., Milwaukee, WI, USA). Recordings were made at a paper speed of 50 mm/s, with a gain of 20 mV/10 mm and a digital filter of 0.05–150 Hz. ECG analysis included heart rate, R-wave voltage in standard lead II, PQ interval (or PR interval when the Q wave was absent), QRS complex duration, QT interval duration, and corrected QT interval (QTc).

On Day 30 of the experiment, blood samples were collected from the right ventricular cavity under general chloral hydrate anesthesia (300 mg/kg) for hematological and biochemical analyses. The rats were euthanized by exsanguination under anesthesia. During necropsy, the general appearance of the internal organs and the presence or absence of fluid in the serous cavities were assessed. Immediately after excision, the wet weights of the brain, liver, both kidneys, spleen, heart, thymus, both adrenal glands, and both testes were measured using an A&D-FX300 balance (A&D, Japan).

The liver, right kidney, heart, thymus, brain, spleen, right testis or ovary, stomach, small intestine, large intestine, and skin at the application site of the test preparation were subjected to histological examination. The organs were fixed in 10% neutral buffered formalin and embedded in paraffin using an AGT-11-FMP carousel-type automatic tissue processor (FIZMEDPRIBOR-M, Moscow, Russia). Sections prepared on a CUT 5062 rotary microtome (SLEE Medical GmbH, Nieder-Olm, Germany) were stained with hematoxylin and eosin using a Leica Autostainer XL (ST5020) histostainer (Leica Biosystems Nussloch GmbH, Nussloch, Germany). Histological examination was performed using a Nikon Eclipse Ni-U biological microscope equipped with a visualization and morphometry workstation (Nikon, Tokyo, Japan) and a Hamamatsu NanoZoomer-SQ laboratory slide scanner (Hamamatsu, Hamamatsu, Japan).

In venous blood serum, biochemical parameters commonly used in toxicology as markers of internal organ damage were determined using a Vitalit 1000 automatic biochemical and immunoturbidimetric analyzer (I.S.E. S.r.l., Rome, Italy), including total serum protein, total cholesterol, creatinine, glucose, urea, and the activities of alanine aminotransferase (ALT) and aspartate aminotransferase (AST). Hemoglobin concentration, erythrocyte count, leukocyte count, leukocyte differential (granulocytes, lymphocytes, and monocytes), and platelet count were determined using a DREW-3 automated hematology analyzer (Drew Scientific, Plantation, FL, USA) with D3-PAC diagnostic reagents (Drew Scientific, USA).

The toxic effects of the preparations were assessed based on changes in the clinical condition of the animals, instrumental examination findings (ECG), hematological and biochemical blood parameters, and the morphohistological characteristics of the internal organs of animals receiving the prototype nanomedicine in comparison with the control group. For objective data analysis, the specialists who performed histological and hematological examinations were blinded.

### 4.6. Statistical Analysis

The statistical program SPSS 25.0 (IBM, Armonk, NY, USA) was used to process the results of our biomedical examinations. The Shapiro–Wilk and Kolmogorov–Smirnov criteria were used to evaluate the normality of indicator distributions. For descriptive statistics of continuous quantitative indicators, which obeyed the law of normal distribution the Mean (M), Std. Deviation (±σ), Std. Error, 95% Confidence Interval for Mean (95CI), Minimum, Maximum were used, and for indicators, which did not follow the law of normal distribution, the median and quartiles were used.

In the cell experiments, the mean value was determined in the control group. Relative to the mean value in each experiment, the percentages in the studied groups were calculated, obtaining the final figure, which was the percentage of the control. For the comparative analysis of multiple subgroups, one-factor ANOVA analysis was performed using post hoc comparisons and Dunnett’s test (for comparison with controls). Independent samples that did not follow the normal distribution were compared using the Kruskal–Wallis test, and the Bonferroni correction for multiple comparisons was taken into account when calculating the *p*-value. For all criteria and tests, differences were considered statistically significant at *p* < 0.05.

The results obtained in the animal studies were statistically analyzed by calculating the arithmetic Mean (M), Std. Deviation (±σ). Since the data were normally distributed, parametric statistical methods were used for further analysis. The statistical significance of differences between groups was assessed by pairwise comparison of the animal groups using Student’s *t*-test. Differences were considered statistically significant at *p* < 0.05.

## 5. Conclusions

Summary of results:

1. The use of citric acid for the preparation of nanocomposites may be promising not only from the standpoint of nanoparticle stabilization and minimizing their size, but also for biomedical applications—ranging from regenerative medicine materials to antibacterial coatings and drug carriers.
2. By the precipitation method using ammonium dihydrogen phosphate from a nitrate solution in the presence of citric acid at various ratios, cerium phosphate–citric acid nanoparticles were obtained, possessing a single-phase CePO_4_ (rhabdophane) structure with a bimodal size distribution (width 3–10 nm, length 15–40 nm, distribution maxima 4–6 nm × 18–23 nm) and a coherent scattering region of 15–25 nm according to TEM and XRD data. Their composite nature was confirmed by IR spectroscopy (presence of coordinated citric acid; the predominant coordination mode is monodentate (bridging) via oxygen atoms of terminal carboxyl groups of citric acid).
3. The crystallite size initially increases with increasing citric acid content and then decreases with further addition. Thermal treatment of the samples (200 °C, 2 h) leads to significant removal of citric acid; however, it does not improve nanoparticle morphology and therefore represents an unnecessary stage of production.
4. A statistically significant dose-dependent antioxidant activity was established for Ce:3CA, Ce:1CA, and Ce:0.5CA samples, most pronounced for Ce:0.5CA, which exceeded the antioxidant potency of ascorbic acid. In contrast, Ce:5CA demonstrated pro-oxidant activity across all concentrations, with maximal expression at 10^−3^ mol/L.
5. In vitro biomedical studies confirmed the biocompatibility (non-cytotoxicity to human fibroblasts) of the nanocomposite samples. The Ce:0.5CA sample showed the greatest efficacy, demonstrating the strongest stimulation of fibroblast metabolism. Only Ce:5CA at the high concentration caused inhibition of cellular metabolism and proliferation, consistent with its pro-oxidant effect.
6. Comprehensive analysis identified Ce:0.5CA as the most promising nanocomposite for further development of a regenerative drug. The second preferred sample was Ce:1CA.
7. Toxicological studies in different animal models confirmed the safety of the selected nanoproduct prototype, including histomorphological analysis of internal organs. Ce:0.5CA sol (10^−3^ M) showed no toxic effects after single oral administration at the maximum possible dose of 50 mL/kg, after single dermal application on intact skin at 10 mL/kg, or after daily application for one month on a model of full-thickness acute skin wounds at 2 mL/kg.
8. The proposed synthesis approach for cerium phosphate–citrate nanocomposites enable reproducible production of citrate-modified CePO_4_ nanoparticles with controlled morphology and useful biomedical properties. From a manufacturing perspective, the method is simple, easily reproducible, environmentally friendly, and does not require significant financial or time resources.

Thus, optimal conditions for the synthesis of morphologically uniform nanocomposites of the CePO_4_–CA system with rhabdophane-type nanocrystals of approximately 5 × 20 nm were established. The most promising material for biomedical applications was identified as Ce:0.5CA, which exhibits the highest regenerative and antioxidant potential and has been proven safe in different animal species under dermal and intragastric administration, as well as in wound models. This supports the potential application of such nanocomposites in cosmetology and regenerative medicine.

## 6. Patents

Patent for invention No. 2855620 “Method for producing phosphate-containing compositions based on cerium” (state registration date 2 February 2026), and patent for invention No. 2863158 “Method for producing a nanosized phosphate-containing composition based on cerium (registration date 29 May 2026) were obtained.

## Figures and Tables

**Table 1:** FTIR absorption bands and assignments for the investigated samples.

Characteristic Absorption Bands (cm^−1^)	Assignment of FTIR Absorption Bands							
	Ce:5CA (Non-Calcined)	Ce:3CA (Non-Calcined)	Ce:1CA (Non-Calcined)	Ce:0.5CA (Non-Calcined)	Ce:5CA (Calcined)	Ce:0.5CA (Calcined)	Citric Acid	Bare CePO_4_
O–H stretching vibrations of hydrogen-bonded hydroxyl groups	3448	3439	3445	3441	3458	3453	3268	3432
Carbonyl (C=O) stretching vibrations	1714	1713	1714	1714	1706		1753	
Carbonyl stretching vibrations/overtone of O–H vibrations	1625	1626	1626	1626	1624	1627	1686	1628
CH_2_–CO group vibrations	1435	1426	1433	1433	1426		1418	
Skeletal C–C stretching vibrations	1328 1337	1327 1337	1328 1235	1237 1239	1338 1328		1290 1208 1170 1107	
Asymmetric P–O stretching vibrations in phosphate anions	1001	1003	999	1002	999	999		1002
Out-of-plane O–H bending vibrations	669	668	667	668	665		640	
P–O bending vibrations	613	612	613	614	614	614		612

**Table 2:** Differences in the mean chemiluminescence (ChL) curve area and maximum chemiluminescence intensity of the analyzed cerium orthophosphate–citrate nanocomposite samples compared with the control and with ascorbic acid (reference antioxidant).

Sample, Concentration	Max (Average ± Std. Dev.), kPPS	*p* _(max)_	S (Average ± Std. Dev.), kPPS·s	*p* (S)
Control	6.85 ± 0.33		78.16 ± 1.71	
Ascorbic acid, 10^−3^ M	3.81 ± 0.03	*	64.54 ± 2.07	*
Ascorbic acid, 10^−4^ M	4.65 ± 0.20	*	76.65 ± 0.92	
Ascorbic acid, 10^−5^ M	4.61 ± 0.06	*	77.43 ± 2.94	
Ce:5CA, 10^−3^ M	12.43 ± 0.16	*#	104.14 ± 0.74	*#
Ce:5CA, 10^−4^ M	9.42 ± 0.30	*#	79.57 ± 1.11	*
Ce:5CA, 10^−5^ M	9.07 ± 0.37	*#	78.76 ± 1.69	
Ce:3CA, 10^−3^ M	2.66 ± 0.12	*#	28.84 ± 0.79	*#
Ce:3CA, 10^−4^ M	5.83 ± 0.06		71.63 ± 2.56	
Ce:3CA, 10^−5^ M	6.63 ± 0.06	#	76.57 ± 3.17	
Ce:1CA, 10^−3^ M	2.66 ± 0.12	*#	29.38 ± 1.33	*#
Ce:1CA, 10^−4^ M	6.55 ± 0.31	#	72.71 ± 3.54	
Ce:1CA, 10^−5^ M	6.86 ± 0.09	#	77.40 ± 2.20	
Ce:0.5CA, 10^−3^ M	2.06 ± 0.09	*#	30.27 ± 0.89	*#
Ce:0.5CA, 10^−4^ M	4.77 ± 0.08	*	69.83 ± 2.95	*#
Ce:0.5CA, 10^−5^ M	5.22 ± 0.09	*	76.62 ± 1.51	

Note: * *p* < 0.05 vs. the control; # *p* < 0.05 vs. ascorbic acid at the corresponding concentration.

**Table 3:** Body weight dynamics of rats during the acute dermal toxicity study following dermal application (topical administration) of the investigated medical device prototypes.

Medical Device Prototype, Dose	Sex	Observation Day								
		«0»	«1»	«2»	«3»	«7»	«14»	«21»	«28»	«30»
Water for injection (control); 10 mL/kg	♂	296.7 ± 26.4	307.3 ± 26.4	313.7 ± 25.5	311.0 ± 24.6	321.7 ± 22.7	341.7 ± 32.5	363.0 ± 33.0	386.0 ± 41.6	390.7 ± 41.6
	♀	239.7 ± 18.6	239.3 ± 12.7	241.0 ± 13.1	240.7 ± 14.0	245.7 ± 13.8	252.3 ± 15.0	266.3 ± 19.7	272.0 ± 21.2	274.0 ± 20.2
CePO_4_–CA NPs (10^−3^ mol/L); 10 mL/kg	♂	296.0 ± 15.6	306.7 ± 15.0	314.3 ± 19.9	312.0 ± 16.7	320.0 ± 17.1	337.0 ± 21.0	360.0 ± 18.0	372.0 ± 26.6	385.7 ± 17.0
	♀	243.3 ± 19.1	240.0 ± 23.3	237.0 ± 27.2	237.0 ± 23.3	242.7 ± 17.6	252.3 ± 16.2	254.7 ± 17.5	265.3 ± 19.6	271.3 ± 24.4

**Table 4:** Electrocardiographic parameters and heart rate in rats during the subchronic toxicity study (Mean ± SD).

Group	Sex	Parameter	Observation Day				
			«0»	«7»	«14»	«21»	«28»
Water for injection (control); 2 mL/kg	♂	PQ	43.0 ± 4.8	45.5 ± 4.1	47.0 ± 8.7	51.5 ± 5.7	50.5 ± 6.2
		QRS	19.5 ± 1.0	14.5 ± 3.4	17.0 ± 4.8	18.0 ± 1.6	17.5 ± 1.0
		Heart rate	395.9 ± 26.1	414.4 ± 13.9	418.2 ± 59.1	391.4 ± 33.2	422.7 ± 12.6
		QT	66.0 ± 11.4	66.0 ± 13.4	74.0 ± 12.0	71.0 ± 5.3	66.5 ± 6.6
		QTc	199.3 ± 11.3	201.0 ± 14.1	209.0 ± 11.0	205.0 ± 3.7	202.3 ± 7.5
		R(II)	6.6 ± 1.0	6.5 ± 0.7	5.6 ± 0.9	5.4 ± 2.0	5.4 ± 1.2
	♀	PQ	42.0 ± 5.4	45.5 ± 8.5	48.0 ± 2.8	52.0 ± 7.7	50.0 ± 5.9
		QRS	17.0 ± 3.8	18.0 ± 1.6	15.0 ± 3.5	17.5 ± 1.9	16.0 ± 1.6
		Heart rate	399.0 ± 17.1	420.8 ± 18.0	425.8 ± 21.8	428.3 ± 32.1	425.8 ± 22.5
		QT	71.5 ± 9.8	69.5 ± 11.0	67.5 ± 3.0	74.0 ± 8.3	72.0 ± 6.3
		QTc	206.3 ± 10.1	205.8 ± 10.1	203.8 ± 3.9	210.0 ± 7.3	208.5 ± 6.6
		R(II)	6.1 ± 1.3	5.1 ± 1.4	5.9 ± 1.5	5.4 ± 1.3	5.9 ± 1.1
CePO_4_–CA NPs (10^−3^ M) 2 mL/kg	♂	PQ	42.5 ± 5.0	48.0 ± 9.1	44.5 ± 4.4	49.0 ± 3.5	49.0 ± 6.6
		QRS	19.0 ± 2.0	15.5 ± 4.1	17.5 ± 1.9	18.5 ± 1.9	16.0 ± 2.8
		Heart rate	408.4 ± 16.6	418.9 ± 26.8	412.7 ± 20.2	423.9 ± 23.9	424.5 ± 29.2
		QT	66.5 ± 16.6	64.0 ± 14.0	74.5 ± 7.2	64.5 ± 8.1	72.0 ± 6.7
		QTc	202.3 ± 16.3	200.8 ± 14.4	210.3 ± 6.4	201.5 ± 7.5	209.3 ± 6.3
		R(II)	7.2 ± 2.1	7.4 ± 1.0	7.4 ± 1.4	6.5 ± 0.3	6.3 ± 1.3
	♀	PQ	45.0 ± 5.8	47.3 ± 4.6	48.5 ± 5.7	45.5 ± 3.4	49.5 ± 8.7
		QRS	19.0 ± 2.0	18.7 ± 2.3	13.5 ± 3.4	19.0 ± 1.2	18.5 ± 1.9
		Heart rate	398.9 ± 33.9	419.9 ± 28.2	425.3 ± 26.2	422.2 ± 27.9	421.6 ± 27.3
		QT	76.0 ± 5.7	73.3 ± 5.8	71.0 ± 4.2	70.5 ± 6.0	74.0 ± 5.4
		QTc	210.3 ± 5.4	208.3 ± 4.9	206.8 ± 4.5	208.3 ± 8.5	209.8 ± 5.9
		R(II)	7.0 ± 1.4	6.2 ± 1.3	6.8 ± 0.9	6.4 ± 0.3	6.1 ± 1.1

**Table 5:** Absolute organ weights of rats during the subchronic toxicity study following dermal administration (topical application) of the investigated medical device prototypes (Mean ± SD).

Medical Device Prototype, Dose	Sex	Body Weight of Rats, g	Internal Organ						
			Heart	Brain	Liver	Thymus	Spleen	Kidneys	Testes
Water for injection (control); 2 mL/kg	♂	314.8 ± 6.6	1.07 ± 0.08	1.34 ± 0.19	11.51 ± 1.75	0.36 ± 0.10	1.07 ± 0.14	2.357 ± 0.184	4.00 ± 0.94
	♀	254.5 ± 16.2	0.91 ± 0.06	1.30 ± 0.16	9.08 ± 0.78	0.45 ± 0.20	0.99 ± 0.26	2.081 ± 0.166	
CePO4–CA NPs (10^−3^ M), 2 mL/kg	♂	315.3 ± 14.7	1.02 ± 0.13	1.39 ± 0.05	10.33 ± 0.400	0.44 ± 0.18	1.28 ± 0.27	2.323 ± 0.241	3.89 ± 0.91
	♀	254.3 ± 14.9	0.84 ± 0.11	1.40 ± 0.10	8.88 ± 0.72	0.43 ± 0.12	0.81 ± 0.27	1.972 ± 0.235	

Note: *p* < 0.05 compared with the control group.

**Table 6:** Composition of the solutions used for the preparation of cerium orthophosphate nanoparticle-based composites.

Sample Designation	1:5	1:3	1:1	1:0.5
Mass ratio of reagents	Ce:5CA	Ce:3CA	Ce:1CA	Ce:0.5CA
**Initial solutions**				
Initial concentration of Ce(NO_3_)_3_, mol/L	0.726	0.726	0.726	0.726
Initial concentration of Ce(NO_3_)_3_, g/L (as CeO_2_)	125	125	125	125
Initial concentration of NH_4_H_2_PO_4_, g/L (mol/L)	100/0.752 mol/L	100/0.752 mol/L	100/0.752 mol/L	100/0.752 mol/L
Volume (concentration) of Ce(NO_3_)_3_ (total volume = 1 L)	100 mL (12.5 g/L as CeO_2_)	100 mL (12.5 g/L as CeO_2_)	100 mL (12.5 g/L as CeO_2_)	100 mL (12.5 g/L as CeO_2_)
Volume (concentration) of NH_4_H_2_PO_4_ (total volume = 1 L)	125 mL (25 g/L), 1.5-fold excess	125 mL (25 g/L), 1.5-fold excess	125 mL (25 g/L), 1.5-fold excess	125 mL (25 g/L), 1.5-fold excess
Volume (concentration) of citric acid (total volume = 1 L)	312.5 mL (62.5 g/L)	187.5 mL (37.5 g/L)	67.5 mL (12.5 g/L)	33.75 mL (6.25 g/L)
**Synthesis conditions**				
Solution pH	1–2	2	3	3
Volume of added H_2_O, mL (total volume = 1 L)	462.5	587.5	707.5	258.75
Stirring speed and time	230 rpm, 30 min			

## Data Availability

The original contributions presented in this study are included in the article. Further inquiries can be directed to the corresponding author.
